# Immune response and pathogen invasion at the choroid plexus in the onset of cerebral toxoplasmosis

**DOI:** 10.1186/s12974-021-02370-1

**Published:** 2022-01-13

**Authors:** Caio Andreeta Figueiredo, Johannes Steffen, Lorena Morton, Sushmitha Arumugam, Oliver Liesenfeld, Mária A. Deli, Andrea Kröger, Thomas Schüler, Ildiko Rita Dunay

**Affiliations:** 1 Institute of Inflammation and Neurodegeneration, Medical Faculty, Otto-Von-Guericke University Magdeburg, Magdeburg, Germany; 2 Institute for Microbiology and Hygiene, Charité Medical School, Berlin, Germany; 3 Institute of Biophysics, Biological Research Centre, 6726 Szeged, Hungary; 4 Institute for Medical Microbiology and Hospital Hygiene, Medical Faculty, Otto-Von-Guericke University Magdeburg, Magdeburg, Germany; 5 Institute of Molecular and Clinical Immunology, Medical Faculty, Otto-Von-Guericke University Magdeburg, Magdeburg, Germany; 6 Center for Behavioral Brain Sciences, CBBS, Magdeburg, Germany

**Keywords:** *Toxoplasma gondii*, Neuroinflammation, Choroid plexus, Blood–CSF barrier, Blood–brain barrier, Tight junctions, Matrix metalloproteinases

## Abstract

**Background:**

*Toxoplasma gondii* (*T. gondii*) is a highly successful parasite being able to cross all biological barriers of the body, finally reaching the central nervous system (CNS). Previous studies have highlighted the critical involvement of the blood–brain barrier (BBB) during *T. gondii* invasion and development of subsequent neuroinflammation. Still, the potential contribution of the choroid plexus (CP), the main structure forming the blood–cerebrospinal fluid (CSF) barrier (BCSFB) have not been addressed.

**Methods:**

To investigate *T. gondii* invasion at the onset of neuroinflammation, the CP and brain microvessels (BMV) were isolated and analyzed for parasite burden. Additionally, immuno-stained brain sections and three-dimensional whole mount preparations were evaluated for parasite localization and morphological alterations. Activation of choroidal and brain endothelial cells were characterized by flow cytometry. To evaluate the impact of early immune responses on CP and BMV, expression levels of inflammatory mediators, tight junctions (TJ) and matrix metalloproteinases (MMPs) were quantified. Additionally, FITC-dextran was applied to determine infection-related changes in BCSFB permeability. Finally, the response of primary CP epithelial cells to *T. gondii* parasites was tested in vitro.

**Results:**

Here we revealed that endothelial cells in the CP are initially infected by *T. gondii*, and become activated prior to BBB endothelial cells indicated by MHCII upregulation. Additionally, CP elicited early local immune response with upregulation of IFN-γ, TNF, IL-6, host-defence factors as well as swift expression of CXCL9 chemokine, when compared to the BMV. Consequently, we uncovered distinct TJ disturbances of claudins, associated with upregulation of MMP-8 and MMP-13 expression in infected CP in vivo, which was confirmed by in vitro infection of primary CP epithelial cells. Notably, we detected early barrier damage and functional loss by increased BCSFB permeability to FITC-dextran in vivo, which was extended over the infection course.

**Conclusions:**

Altogether, our data reveal a close interaction between *T. gondii* infection at the CP and the impairment of the BCSFB function indicating that infection-related neuroinflammation is initiated in the CP.

**Supplementary Information:**

The online version contains supplementary material available at 10.1186/s12974-021-02370-1.

## Background

Toxoplasmosis is a foodborne parasitic disease caused by the obligate intracellular protozoan *Toxoplasma gondii* (*T. gondii*). It is estimated that more than one-third of the world’s human population is infected with *T. gondii* and its seroprevalence increases gradually with age [1–3]. Following uptake via oral ingestion of contaminated food or water, the parasites proliferate within a variety of nucleated cells, infect circulating leukocytes, successfully cross all the barriers of the body and spread throughout host tissues [4]. Despite of the central nervous system (CNS) being an immune privileged site shielded from peripheral infections and inflammation, *T. gondii* parasites are able to invade the CNS. Previous studies have proposed different mechanisms of parasite invasion, including: (1) active paracellular migration of free parasites; (2) transmigration of hypermotile infected leukocytes, defined as “Trojan horse” mechanism, and (3) infection and replication of parasites within brain endothelial cells [5–12]. Once *T. gondii* crosses the brain biological barriers, like the blood–brain barrier (BBB), they invade brain-resident cells and persist in cysts lifelong [13–15]. The cyst formation within neurons develops a stress-mediated response followed by ongoing basal neuroinflammation, which leads to altered neuronal function, and potentially to behavioral alterations and neuropsychiatric diseases [16–19]. In immunodeficient individuals, the infection can lead to disruption of tissue cysts and uncontrolled parasite proliferation, resulting in toxoplasma encephalitis (TE) [2, 20, 21].

Several studies have demonstrated the decisive involvement of the BBB in the invasion of *T. gondii* and the ensuing development of neuroinflammation [22, 23]. In fact, the choroid plexus (CP) is another barrier and potential interface for pathogen invasion into CNS. The CP is the main structure forming the blood–CSF barrier (BCSFB), and is crucial for CNS homeostasis and cerebrospinal fluid (CSF) secretion. Located within the four brain ventricles, the CP is a villous and selective organ formed by adjacent epithelial cells anchored to a basal lamina and an inner core of resident immune cells surrounding a dense vascular network of fenestrated endothelial cells. Choroidal epithelial cells are tightly interconnected by tight junction proteins (TJ), and control the molecular and cellular composition of the CSF [24, 25]. The ability of this unique neuro-immune interface to actively integrate signals between brain and periphery is fundamental to CNS immunity [26], in which CP regulation of immune cell trafficking is considered a central point in the initiation of inflammatory brain responses [27]. Alterations or even disruption of the CP epithelium in response to stressful events have detrimental effects on barrier permeability compromising the BCSFB functions [28]. Indeed, BCSFB breakdown has been implicated in neurodegenerative diseases [29–33] and during infection-induced inflammation [31, 34], often indicating the involvement of matrix metalloproteinases (MMPs). Moreover, recent studies have described that the BCSFB serves as a hotspot for direct pathogen infiltration into the CNS [35]. However, data concerning the contribution of the CP to *T. gondii* invasion, and subsequent neuroinflammation are controversial and incomplete. For example, the analysis of postmortem samples from immunodeficient patients with cerebral toxoplasmosis identified the CP as a site of infection [36]. On the contrary, models of reactivated TE indicate no evidence for the involvement of CP in systemic parasitic dissemination [37, 38].

Here, we demonstrate that, prior to the activation of endothelial cells in the BBB, parasites invading the CNS rapidly seize choroidal endothelial cells. As a result, a prompt immune response is initiated in the CP as shown by the upregulation of cytokines, chemokines, and host defense factors, followed by the expression of MMPs, TJ disturbance of CP epithelial cells, and subsequent increased barrier permeability. Together, our results show that the infection takes place in the CP, and indicate sudden functional impairment of the BCSFB upon the onset of the CNS invasion by *T. gondii*.

## Methods

### Mice and infections in vivo

Experiments were conducted with female C57BL/6 J mice (8–14 weeks old, purchased from Janvier, Cedex, France). All animals were group-housed in a 12-h day/night cycle at 22 °C with free access to food and water under specific pathogen-free conditions and according to institutional guidelines approved by the Animal Studies Committee of Saxony-Anhalt. In order to investigate early *T. gondii* infection, mice were infected by intraperitoneal (*i.p.*) injection of either cysts or tachyzoites. For cyst infection, 2 cysts of the type II ME49 strain harvested from the brains of female NMRI mice infected *i.p.* with *T. gondii* cysts 6–12 months earlier were used as previously described [39]. For tachyzoites infection, type II *T. gondii* reporter parasites of the PTG-GFPS65T strain were grown in monolayers of human foreskin fibroblast (HFF) with DMEM medium (FG0435, Biochrom, Germany), supplemented with 10% fetal bovine serum (FBS) (ThermoFisher, Germany), 1% penicillin/streptomycin (Pen/Strep; Sigma, USA) and 1% non-essential amino acids (NEEA) (ThermoFisher, Germany) as previously described [40]. Freshly egressed parasites were filtered through a 5 μm Millex-SV syringe filter (Millipore, Germany), and the number of living tachyzoites determined by counting under a bright-field microscope using Trypan Blue 0.4%. Mice were infected *i.p.* with 1 × 10^5^ reporter parasites in 200 µl PBS.

### Organ isolation

Mice were deeply anaesthetized by isoflurane inhalation (Baxter), the CSF was collected and thereafter animals were transcardially perfused with 60 ml PBS. For immunofluorescence samples, perfusion was additionally done with 20 ml of 4% paraformaldehyde (PFA) in PBS. Brain, spleen and spinal cord were removed and stored in sterile ice-cold PBS or RNAlater (Sigma) for further processing. Samples stored in RNAlater were kept at 4 °C overnight and afterwards transferred to − 80 °C.

### Cerebrospinal fluid collection

CSF was collected by the *cisterna magna* puncture technique as described elsewhere [41]. In short, deeply anaesthetized animals were immobilized in a prone position with the head forming a 135° angle with the body, and a sagittal incision of the skin was made inferior to the occiput. Using a stereomicroscope (Stemi 305; ZEISS), the subcutaneous tissue and muscles were dissected, and a glass capillary tube was introduced into the *cisterna magna* through the dura matter, lateral to the arteria *dorsalis spinalis*. An average of 15 µl of CSF per animal were collected, and kept on ice until further processing. Samples were macroscopically assessed for blood contamination, and discarded when contamination was detected.

### Choroid plexus and brain tissue isolation

Isolated brains were placed under a stereomicroscope in dissection buffer containing HBSS (ROTI®Cell, Roth), and 10 mM HEPES (Gibco, ThermoFisher). CPs were isolated from the lateral, third and fourth brain ventricles, and were either processed for total RNA/DNA isolation, or placed in digestion buffer for further cell isolation. Cerebellum, olfactory bulbs, and adjacent brain meninges were removed and discarded. The remaining brain tissue was used for cell isolation followed by flow cytometric analysis, or further utilized for brain microvessels isolation.

### Brain microvessels isolation

Brain microvessels were isolated as previously described [42] with a few modifications, and used for immunofluorescence, or total RNA/DNA isolation. Briefly, brain hemispheres were minced with a scalpel, and homogenized in digestion buffer (HBSS, with 6.75 g/l glucose, 20 mM HEPES) containing 1 mg/ml DNAse I. After incubation (10 min, 37 °C), homogenate was washed in FACS buffer (PBS w/o Ca/Mg, 2 mM EDTA, 2% v/v FBS, 10 mM HEPES). The resultant pellet was resuspended and separated by successive centrifugations in 20% (w/v) bovine serum albumin (BSA)-DMEM/F12 solution. To remove remaining myelin debris, the pellet containing microvessels was resuspended in PBS, fractioned on 22% (v/v) Percoll® (Sigma, #GE17089101) gradient solution and centrifuged for 10 min, 600 × g, w/o brake. The microvessels pellet was recovered and extensively washed in PBS/HEPES.

### Cell isolation

Isolated CP, the remaining brain tissue, liver and lymph nodes (inguinal and mesenteric) were further processed in order to obtain single cell suspensions. CP samples were incubated (20 min, 37 °C) in digestion buffer (PBS w/ Ca/Mg, 2% v/v FBS, 10 mM HEPES) containing 1 mg/ml DNAse I (Sigma, #DN25) and 1 mg/ml Collagenase/Dispase (Sigma, #11097113001). Digested tissues were mechanically dissociated using syringes connected to 22- and 26G needles, then cells were washed with FACS buffer, and used for further analysis. For the isolation of brain cells, liver and lymph nodes, tissues were minced with a scalpel, and homogenized in digestion buffer (HBSS, with 6.75 g/l glucose, 20 mM HEPES) containing DNAse I and Collagenase/Dispase as previously mentioned. Homogenate was incubated (40 min, 37 °C, 200 rpm), and filtered through a 70-μm cell strainer (Falcon®, #352350). The cell suspension was centrifuged (400 × g, 10 min, 4 °C), and the cell pellet separated in a 25–70% discontinuous Percoll® gradient for 20 min without brake. Cells were recovered from the gradient interface, washed with FACS buffer, and used for further analysis.

### Flow-cytometric analysis

Cells were resuspended in FACS buffer, and stained as previously described [39, 40]. In short, cells were incubated with an anti-mouse CD16/32 unconjugated antibody (clone 93, BioLegend) and stained with fixable viability dye Zombie NIR (BioLegend), for 20 min at 4 °C. Subsequently, cells were stained (30 min, 4 °C) using fluorochrome-conjugated antibodies. CD11b (PerCP-Cy5.5, clone M1/70) purchased from eBioscience, CD45 (BV510, clone 30-F11), MHCII (BV711, clone M5/114.15.2), VE-cadherin (BV421, clone BV13), and gp38 (AF488, clone PMab-1) purchased from BioLegend. CD31 (APC, clone MEC13.3) purchased from BD Biosciences. Cells were washed (400 × g, 5 min 4 °C) with FACS buffer, fixed in 4% PFA for 15 min at 4 °C and re-suspended in FACS buffer. For CP, cells were additionally permeabilized with eBioscience™ Permeabilization buffer (Invitrogen), incubated with antibody rabbit-anti-TTR (Abcam) for 40 min, and stained with secondary antibody anti-rabbit AF488 (ThermoFisher). Cells were acquired using Attune NxT Flow Cytometer (ThermoFischer). Data were analyzed using FlowJo software (version 10.5.3, FlowJo LLC, OR, USA).

### FITC-dextran permeability assay

BCSFB and BBB permeability were measured as previously described [34] with modifications. In short, 4 kDa FITC-dextran (Sigma, #46944) was diluted in PBS, and administered intravenously (*i.v.*) at 75 mg/kg body weight mice, 30 min before CSF and brain collection. CSF was collected through the *cisterna magna* as previously described, diluted 100-fold in PBS, and spun down (1000 × g, 5 min). The resulting supernatant was further used for analysis. After perfusion, isolated brains were weighted, minced with a scalpel, homogenized in formamide (Roth) at 0.8 ml per 100 mg tissue, and incubated overnight (37 °C, 200 rpm). Homogenates were spun down (12,000 × g, 5 min), and supernatants were collected and diluted twofold in PBS. All samples were measured in triplicates at λex/λem = 485/520 nm using SpectraMax M5e (Molecular Devices LLC).

### Choroid plexus epithelial cells and *T. gondii* in vitro infection

Primary CP epithelial cell culture was obtained as previously described [43, 44] with modification. Isolated CP from 9 to 14 mice, one-week-old, were used in each preparation. Tissues were pooled in digestion buffer (PBS w/ Ca/Mg, 2% v/v FBS, 10 mM HEPES) containing 1 mg/ml DNAse I and 2 mg/ml Collagenase/Dispase, and incubated (20 min, 37 °C). Digested tissues were mechanically dissociated and cells were washed (400 × g 5 min) with FACS buffer (PBS w/o Ca/Mg, 2 mM EDTA, 2% v/v FBS, 10 mM HEPES). Cell pellet was resuspended, and cultivated with complete medium (DMEM/F12, supplemented with 10% FBS, 1% penicillin/streptomycin, 1% ITS (insulin–transferrin–selenite), 40 mg/ml human-EGF (epidermal growth factor, PeproTech, Germany, #AF-100–15). Cells were grown until confluence, for approximately 7 to 10 days, in 12 well plates previously coated with poly-l-lysine (PLL), or on top of PLL-coated coverslips. Once confluent, cells were infected at multiplicity of infection (MOI) = 5 with type II PTG-GFPS65T tachyzoites, and incubated at 37 °C. After 6 h, culture medium was removed, cells were washed with PBS, and further used for total RNA/DNA isolation, flow cytometric analysis (validation) or immunofluorescence. Naïve controls were treated with pre-warmed fresh medium.

### Immunofluorescence

Immunofluorescence staining was performed for CP whole mount, BMV mount, brain sections and cell culture coverslips. For brain sections, isolated brains were post-fixed (4 h, at 4 °C), soaked in 30% sucrose in PBS (2 days, at 4 °C), and frozen in cryo media (OCT Compound, Tissue Tek). Coronal sections (20 µm) were obtained (Thermo Scientific CryoStar NX50) and only sections containing CP were stained. BMV mount, brain sections and coverslips were stained directly on glass slides. CP whole mount staining were performed utilizing a free-floating approach. All samples from different origins were stained and mounted with the same protocol unless otherwise stated. In short, samples were fixed (4% PFA in PBS, 20 min, 4 °C), washed twice with washing solution (PBS 0.1% (v/v) Triton X-100), and blocked/permeabilized with PBS 0.3% (v/v) Triton X-100 5% normal-goat-serum (NGS) and unconjugated F(ab′)2-goat anti-mouse IgG (H + L) antibody (1:500, Thermo Scientific), for 2 h at 4 °C. Next, samples were incubated with antibody solution (PBS 0.1% (v/v) Triton X-100 2% NGS) containing the primary antibodies of interest: anti-SAG1 (1:50, clone D61S, ThermoFisher), anti-GFP (1:1000, #132004, SYSY), anti-E-cadherin (1:500, #PA5-85088, ThermoFisher), anti-PDGFRβ (1:200, clone R.140.4, ThermoFisher), anti-ZO-1(1:50, clone R26.4C, ThermoFisher), anti-IBA-1(1:500, #234004, SYSY), anti-CD31 (1:50, clone MEC13.3, BioLegend), anti-claudin-2 (1:500, clone MH44, ThermoFisher), VE-cadherin (1:40, clone BV13, ThermoFisher). Primary antibodies were incubated overnight at 4 °C, then samples were washed twice, and incubated (30 min, RT) in antibody solution with secondary antibodies (1:1000) tagged with AF488, AF555, AF594 and AF647 according to each antibody host. Next, samples were washed twice, and mounted with ProLong™ Gold Antifade Mountant with DAPI (ThermoFisher). In some samples, anti-CD45 (clone 30-F11) conjugated antibody was used, and an additional final staining step was added. For brain sections, antigen retrieval (10 mM citrate buffer, pH 6.0, 0.1% Tween-20) was performed at 96 °C for 30 min, before the blocking and permeabilization step. Images were generated using a Leica TCS SP8 microscope, and analyzed using the ImageJ software (ImageJ 1.52p).

### DNA and RNA isolation

Total DNA and RNA were isolated from CP, BMV, spleen, spinal cord, brain and CP epithelial cell culture. Samples from CP, BMV and CP epithelial cell culture were isolated with Quick-DNA/RNA Miniprep kit (Zymo Research, Germany) according to the manufacturer’s instructions. Brain, spinal cord, and spleen samples were first homogenized with TriFast (Peqlab, 30-2010) using tubes containing Zirconium oxide beads (Precellys, P000926-LYSK0-A) in a BeadBug 6 homogenizer (Biozym). DNA was isolated from the homogenate according to the manufacturer’s instructions. RNA was isolated from the homogenate by isopropanol precipitation or using peqGOLD total RNA kit (Peqlab) following the manufacturer’s instructions. The concentration and purity of DNA and RNA samples were determined using NanoDrop 2000 spectrophotometer (ThermoFisher; Germany), and samples were stored at − 80 °C until further use.

### qPCR

Parasite burden was assessed in triplicates using 40 ng of isolated DNA, FastStart Essential DNA Green Master and LightCycler® 96 System (both Roche, Germany), as previously described [40]. Thermal-cycling parameters were set as follows: initial activation (95 °C, 10 min), 55 amplification cycles consisting of denaturation (95 °C, 15 s), annealing (60 °C, 15 s) and elongation (72 °C, 15 s). The DNA target was the published sequence of the highly conserved 35-fold-repetitive B1 gene of *T. gondii* [45, 46]. Murine argininosuccinate lyase (*Asl*) was used as reference gene for normalization and relative DNA levels were determined by the ratio *gene of interest/reference gene* and subsequently normalized to mean values of control group [47]. Primers were synthetized by Tib MolBiol (Germany) and used at 200 nM final concentration. Primer sequences are described elsewhere [see Additional file 1].

### RT-qPCR

Gene expression levels of cytokines, inflammatory mediators, host-defense factors, tight junctions and MMPs were assessed in triplicates using 20 ng total RNA, TaqMan® RNA-to-CT™ 1-Step Kit (Applied Biosystems, Germany) and LightCycler® 96 (Roche, Germany) as previously described [40]. Thermal-cycling parameters were set as follows: reverse transcription (48 °C, 30 min), inactivation (95 °C, 10 min) followed by 55 cycles of denaturation (95 °C, 15 s) and annealing/extension (60 °C, 1 min). Utilized TaqMan® Gene Expression Assays (Applied Biosystems, Germany) are listed elsewhere [see Additional file 1]. *Hprt* was chosen as *reference gene* and relative mRNA levels were determined by the ratio *gene of interest/reference gene* and subsequently normalized to mean values of control group.

For the genes analyzed using SYBR Green technology, Power SYBR® Green RNA-to-CT™ 1-Step Kit (Applied Biosystems, Germany) was used. Samples were analyzed in triplicates (20 ng of isolated mRNA per reaction) using LightCycler® 96 with the following parameters: reverse transcription (48 °C, 30 min), inactivation (95 °C, 10 min) followed by 55 cycles of denaturation (95 °C, 15 s) and annealing/extension (60 °C, 1 min) and melting curve analysis. The primer sequences are listed elsewhere [see Additional file 1] and were synthetized by Tib MolBiol and used at 100 nM final concentration. *Hprt* was chosen as reference gene and relative mRNA levels were determined by the ratio *gene of interest/reference gene* and subsequently normalized to mean values of control group.

### Statistical analysis

Results were statistically analyzed using GraphPad Prism 7 (GraphPad Software Inc., USA), *post-test* corrections were applied according to software recommendations. Statistical significance was set to *p* ≤ 0.05. All data are presented as arithmetic mean and standard error of the mean (SEM) and are representative of at least two independent experiments. For parasite burden analysis, normalized data were analyzed by multiple *t*-test, with Holm–Šidák correction for multiple comparisons. For flow cytometric analyses, normalized data were analyzed by one-way ANOVA, with Tukey’s correction. For RT-qPCR, data were analyzed by one-way ANOVA followed by Dunnett’s correction. BCSFB permeability assay was analyzed by multiple *t*-test to compare between BCSFB and BBB, and one-way ANOVA followed by Dunnett’s correction was used to compare differences between day 0 and the time-point analyzed from the same barrier type. RT-qPCR data from primary cell culture were analyzed by Student’s *t*-test.

## Results

### Choroid plexus is infected by *T. gondii* upon the invasion

**Fig. 1:**
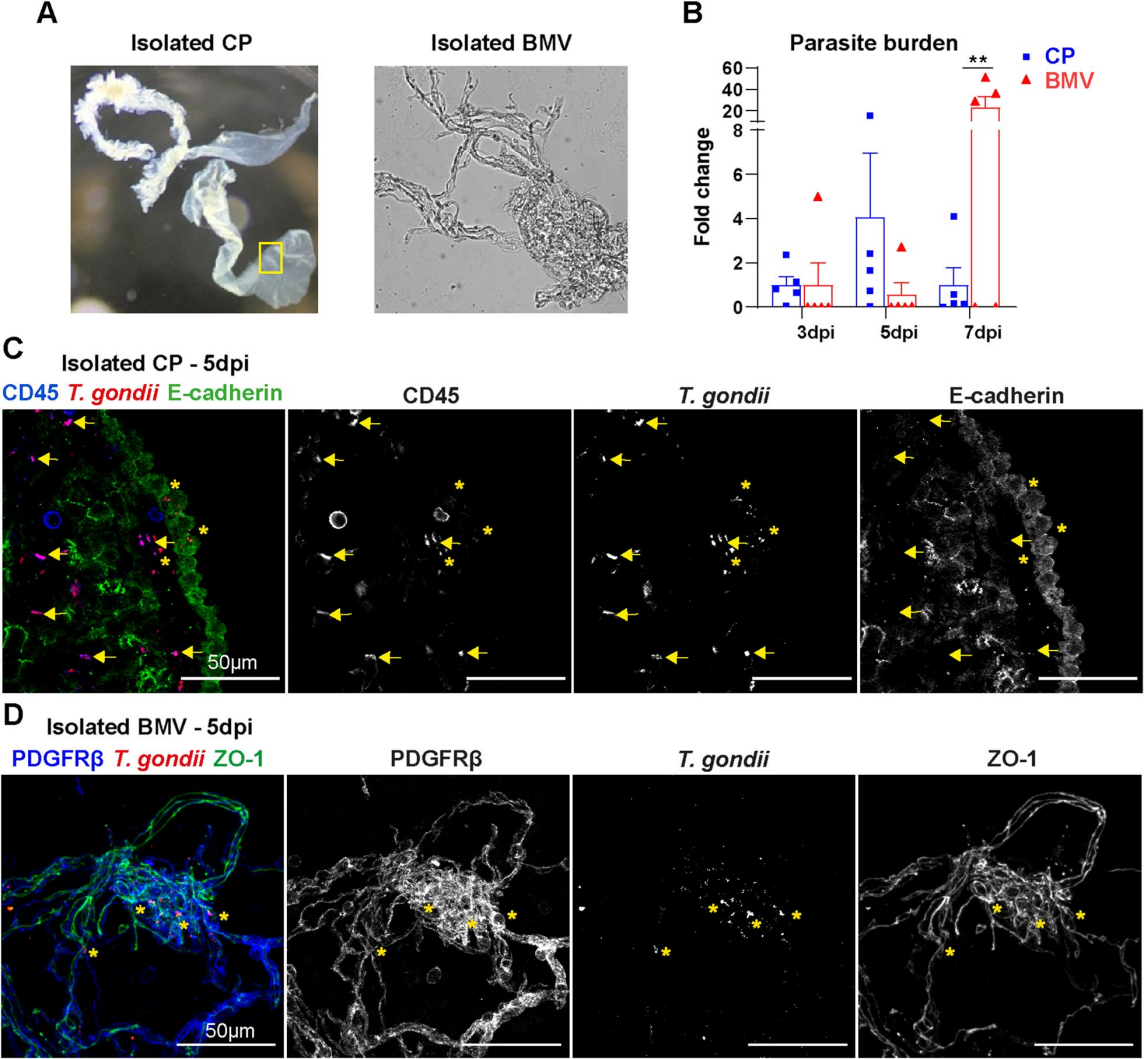
Detection of *T. gondii* in the CP. Mice were infected *i.p.* with 2 cysts of *T. gondii* type II ME49 and samples were collected at 5dpi. From brains, CPs were isolated under stereomicroscope, and the remaining brain tissue was processed for isolation of BMVs. **A** Representative image of freshly isolated CP and a phase-contrast image of isolated BMV. **B** Parasite burden qPCR analysis of isolated CPs (blue) and BMVs (red) from the correlated animals, which were infected at the same time, and analyzed at 3, 5 and 7dpi. The analysis was performed based on the presence of B1 gene of *T. gondii* (*TgB1*) normalized to the murine gene *Asl*. Data were normalized to the mean values of 3 dpi, and bar charts show individual values of a representative experiment, and mean + SEM. *n* = 5 per group. Statistical analysis was performed by multiple *t*-test, with Holm–Šidak correction for multiple comparisons. **p < 0.01. **C** CP whole mount staining to identify immune cells (CD45, blue), *T. gondii* (SAG1, red) and epithelial cells (E-cadherin, green). Scale bars = 50 µm **D** BMVs immune-staining to identify pericytes (PDGFRβ, blue), *T. gondii* (SAG1, red), and the tight junction ZO-1 (green). Yellow arrows indicate the co-localization of parasites with CD45 ^+^ immune cells, and yellow asterisks *T. gondii* signal alone. Scale bars = 50 µm

BCSFB is a gateway to the CNS for different pathogens as well as immune cells under certain inflammatory conditions [26, 35, 48–50]. Assuming that the CP is involved in the parasite entry and early immune response upon the invasion of the brain by *T. gondii*, we investigated the presence of parasites in the CNS through the course of infection. As BBB endothelial cells were shown previously to be directly infected by *T. gondii* [7], we isolated brain microvessels (BMV) that contain endothelial cells, and compared them to isolated CP (Fig. 1A) for parasite burden over time by PCR. Our results revealed the early presence of parasites on CP at 3 dpi, which was increased by day 5 and reduced by day 7 (Fig. 1B). In the BMV, the parasite burden only strongly increased by day 7 (Fig. 1B). Next, CP and BMV of infected animals were isolated and immuno-stained as a three-dimensional whole mount preparation. Within the CP at 5 dpi, we detected parasites (SAG1 ^+^) co-localized with immune cells (CD45^+^) (Pearson’s coefficient, *r* = 0.57), and also extracellular parasites present in the paracellular and stromal regions between the CP epithelial cells (E-cadherin^ +^) without co-localization with E-cadherin (Pearson’s coefficient, *r* = 0.013) (Fig. 1C). In the BMV, parasites were found to be associated to the microvasculature, although no co-localization were detected for PDGFRβ ^+^ pericytes (Pearson’s coefficient, *r* = 0.099) and only a few for ZO−1^+^ endothelial cells (Pearson’s coefficient, *r* = 0.112) (Fig. 1D), which constitute the main components of cerebral microvasculature forming the BBB [51]. Overall, parasites were found in the CP, and apparently more frequently in initial infection at the BCSFB interface than the BBB. Immuno-staining of isolated BMV confirmed the presence of *T. gondii* tachyzoites in BMV at 7 dpi, as detected previously by PCR [see Additional file 2]. Spleens were used as controls to monitor the parasite burden levels in the periphery, indicating the usual spread of the parasites throughout tissues [see Additional file 2]. Parasite burden was just detected starting at 7 dpi in both brain parenchyma (after CP removal) and spinal cord, evidencing the early infection through the CP and the microvasculature [see Additional file 2].

### Choroid plexus endothelial and immune cells are targeted by *T. gondii*

**Fig. 2:**
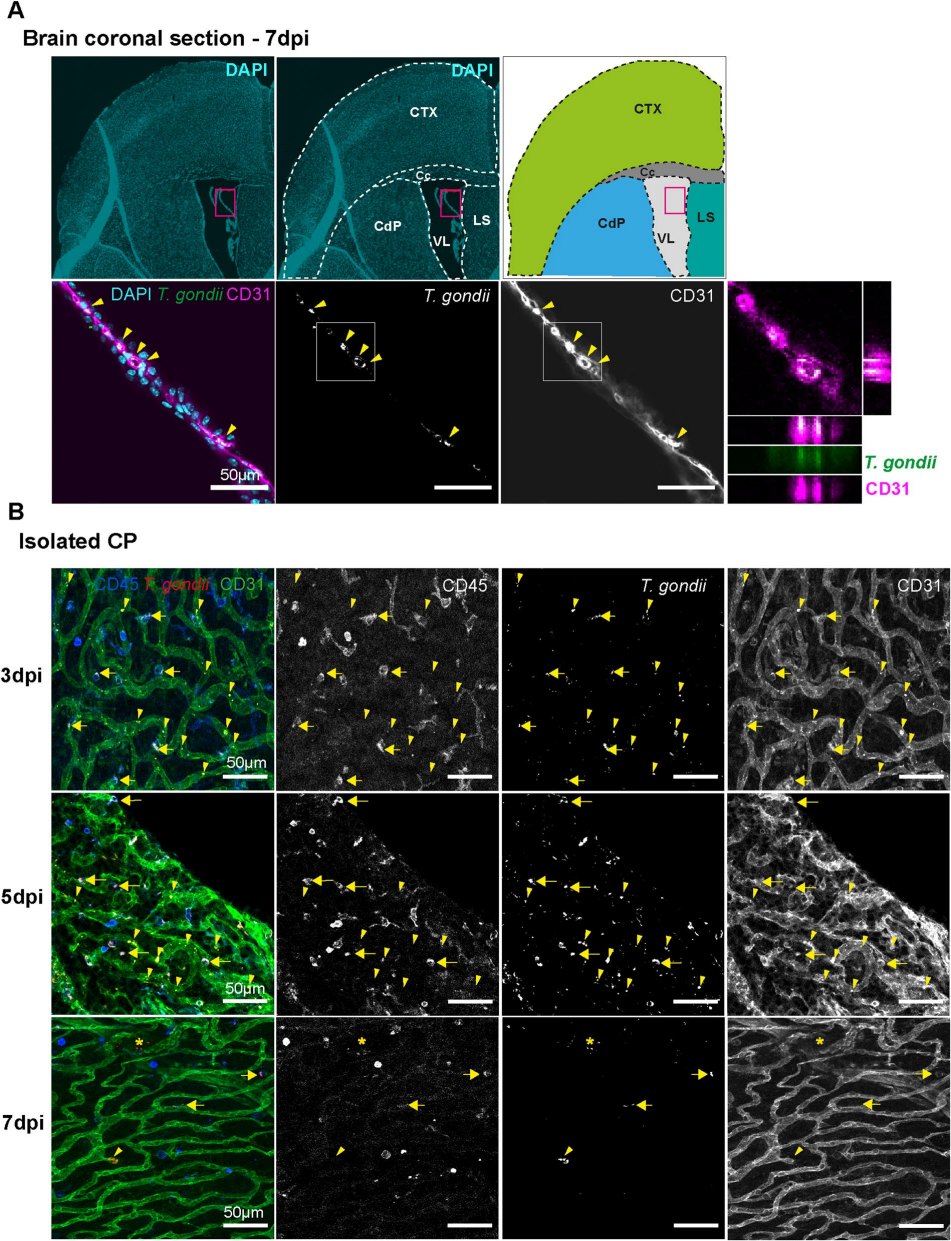
*Toxoplasma gondii* infection of endothelial and immune cells in the CP. **A** Animals were infected *i.p.* with 1 × 10^5^ *T. gondii* type II PTG-GFP tachyzoites. The brains were isolated at 7 dpi, and coronal sections were immune-stained with anti-GFP (green), anti-CD31 (magenta) and DAPI (cyan). First row indicate the location of the CP (magenta rectangle) in the lateral ventricle (VL) according to the mouse brain atlas. Second row magnify the CP area above. Yellow arrowheads indicate the detection of parasites co-localized with endothelial cells (CD31). Scale bars = 50 µm **B** Isolated CPs from animals infected *i.p.* with 1 × 10^5^ PTG-GFP tachyzoites were immune-stained as whole-tissue mount with anti-CD45 (blue), anti-SAG1 (red), and anti-CD31 (green). Animals were infected at the same time, but tissues were analyzed at 3, 5 and 7 dpi. Yellow arrows indicate CD45/SAG1 co-localization, showing immune cells carrying parasites through the BCSFB. Arrowheads indicate co-localization of CD31/SAG1, and asterisks represent SAG1 signal alone. White square indicates magnified area of co-localization signal and orthogonal views of a z-stack analysis. GFP signal plus SAG1 staining were detected in the same channel and colored in red for better visualization. Scale bars = 50 µm. *CTX* cortex, *Cc* corpus callosum, *CdP* caudoputamen, *LS* lateral septal, *VL* lateral ventricle

Given the early presence of *T. gondii* in the CP, we sought to determine the target cell types in this compartment. Therefore, mice were infected *i.p.* with 10 × 10^5^ tachyzoites of the *T. gondii* strain expressing the fluorescent protein GFP (PTG-GFPS65T). First, we analyzed coronal brain sections showing CP from lateral ventricles and found parasites within the choroidal endothelial cells at 7 dpi (Fig. 2A) [and see Additional file 3]. Indeed, at 7 dpi parasites were additionally found in the cortex and the adjacent brain areas [see Additional file 2], simultaneously to the CP [see Additional file 3]. To identify infected immune cells in the BCSFB, isolated CP whole mounts were co-stained for CD45 and CD31. At 3 dpi, we observed *T. gondii* parasites associated to immune cells and endothelial cells in the CP (Fig. 2B). Of note, recruited amoeboid-shape CD45*hi* leukocytes did not appear to be associated to parasites, rather a resident stellate CD45*int* CP macrophages (referred as CP-BAMs (border-associated-macrophages) or epiplexus cells) were co-localized with parasites at this time-point [see Additional file 3]. CP-resident macrophages were also identified by IBA-1 staining and morphology [see Additional file 4]. CP endothelial cells were infected with intracellular parasites at 3 and 5 dpi, but at 7 dpi infected cells were less abundant. Additionally, parasites were also found in the inner core of the CP parenchyma, suggesting the infection of mesenchymal derived stromal cells. Altogether, our results demonstrate the dynamic infiltration of parasites into endothelial and immune cells of the CP in the early phase of infection, confirming our initial data on parasite burden.

### Early immune response at the choroid plexus prior to the BBB

**Fig. 3:**
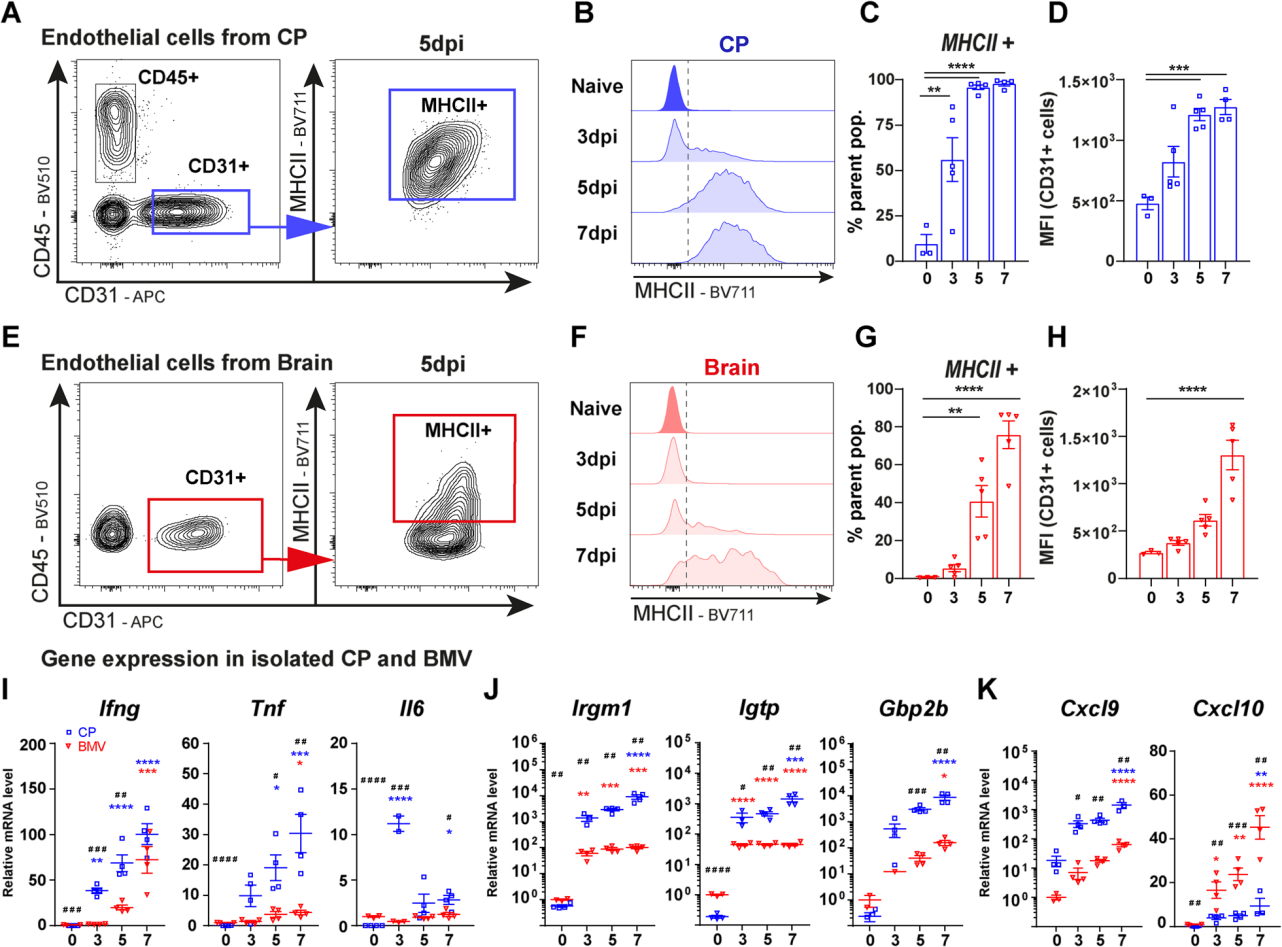
Initial immune response in the CP and BBB upon *T. gondii* infection. Single live cells from (**A**–**D**) isolated CP and (**E**–**H**) brain were analyzed by flow cytometry, and the expression of MHCII was evaluated on endothelial cells (CD31^+^ CD45^−^) from both tissues. Dot plots (**A**, **E**) represent the gating strategy based on FMO controls. Histograms (**B**, **F**) provide a visual representation of the temporal MHCII expression levels by endothelial cells over *T. gondii* early phase of infection. Bar charts (**C**, **G**) show the frequency in % of cells derived from parent population (CD31^ +^ CD45 ^−^). Bar charts (**D**, **H**) show MFI values of MHCII expression on the endothelial cells. RT-PCR of total RNA isolated from CP and BMV was performed for (**I**) cytokine expression, (**J**) IFN-γ-regulated host-defense factors, and (**K**) chemokines. Data from **C**, **D**, **G**, **H** show individual values and mean ± SEM, *n* = 5, *p < 0.05, **p < 0.01, ***p < 0.001, ****p < 0.0001 (one-way ANOVA, with Tukey’s correction). Data from **I**, **J**, **K** were normalized to naïve (day 0) BMV mean, and individual values as mean ± SEM are shown, *n* = 2–4, ^#^*p* < 0.05, ^##^*p* < 0.01, ^###^*p* < 0.001, ^####^*p* < 0.0001, (one-way ANOVA, with Dunnett’s correction). # indicates significant difference between CP and BMV from the same time-point, and * indicates significant difference between day 0 and the time-point being analyzed from the same tissue

Pathogens infecting the CP elicit local immune responses which are associated with the activation of endothelial, epithelial and tissue-resident immune cells [35]. To characterize the early immune response to *T. gondii* in the CP in more details, we first compared the MHCII expression on endothelial cells from CP and remaining brain tissue (Fig. 3A–H). In agreement with the spatiotemporal differences in pathogen distribution (Fig. 1), MHCII upregulation on endothelial cells was first evidenced in the CP followed by later activation of brain endothelial cells (Fig. 3A–H). The definition of endothelial cells as CD45^−^ CD31^+^ was sufficient to identify endothelial cells from both tissues, and nor the additional endothelial marker VE-cadherin neither the fibroblastic marker GP38 (podoplanin) improved the recognition of endothelial cells [see Additional file 5]. The kinetics of MHCII expression by microglia was comparable to brain endothelial cells and reached maximum levels at 7 dpi [see Additional file 6]. Moreover, MHCII expression was analyzed on CP epithelial cells even though less than 10% of the cells express this marker, a significant increase was observed [see Additional file 6]. Of note, total mRNA expression of MHCII (*H2-Aa*) in the CP was also increased during infection [see Additional file 7]. Taken together, choroidal endothelial cells responded earlier to *T. gondii* than brain endothelial cells and microglia, indicating that the CP is a critical interface for early parasite recognition.

To further compare the local inflammatory response in the BCSFB versus the BBB, we analyzed the mRNA expression of pro-inflammatory cytokines which play a central role in host defense upon *T. gondii* infection. Therefore, CP and the BMV were isolated from infected brains and expression of IFN-γ, TNF, and IL-6 were measured (Fig. 3I). Compared to the BMV, CP expression of IFN-γ was higher and earlier elevated. BMV only reached comparable IFN-γ levels at 7 dpi. TNF was significantly higher in the CP at both 5 and 7 dpi and IL-6 levels peaked at 3 dpi and was higher in the CP at 7 dpi (Fig. 3I). We also evaluated the expression of type I interferon-β (IFN-β, *Ifnb1*), which were highly expressed in the CP [see Additional file 7]. Furthermore, the cell-autonomous immune response against intracellular *T. gondii* infection is mediated by IFN-γ-inducible GTPases, designated as host-defense factors (*Irgm1, Igtp, Gbp2b*). Following the elevated IFN-γ levels, we detected higher expression of those genes on the CP compared to the BMV (Fig. 3J). Previous studies have shown specific immune cell trafficking molecules to be upregulated on CP epithelial cells in the presence of IFN-γ and TNF [50]. Accordingly, CXCL9 expression was higher in CP throughout the observation period. On the contrary, CXCL10 was more pronounced in the BMV, and both chemokines were progressively increased during the infection (Fig. 3K). At last, M-CSF (macrophage colony-stimulating factor, *Csf1*) and CX3CL1 (fractalkine), which are chemokines involved in monocyte trafficking across the BCSFB [52] were also found upregulated in the CP at 7 dpi, while no alteration was detected for M-CSF on BMV [see Additional file 7]. Of note, ICAM-1 involved in the general trafficking of immune cells was also upregulated at the CP [see Additional file 7]. Overall, *T. gondii* elicited an early and local immune response at the BCSFB, with the expression of pro-inflammatory cytokines, IFN-γ-inducible GTPases, and leukocyte trafficking molecules.

### Loss of TJ integrity affects BCSFB function in early infection

**Fig. 4:**
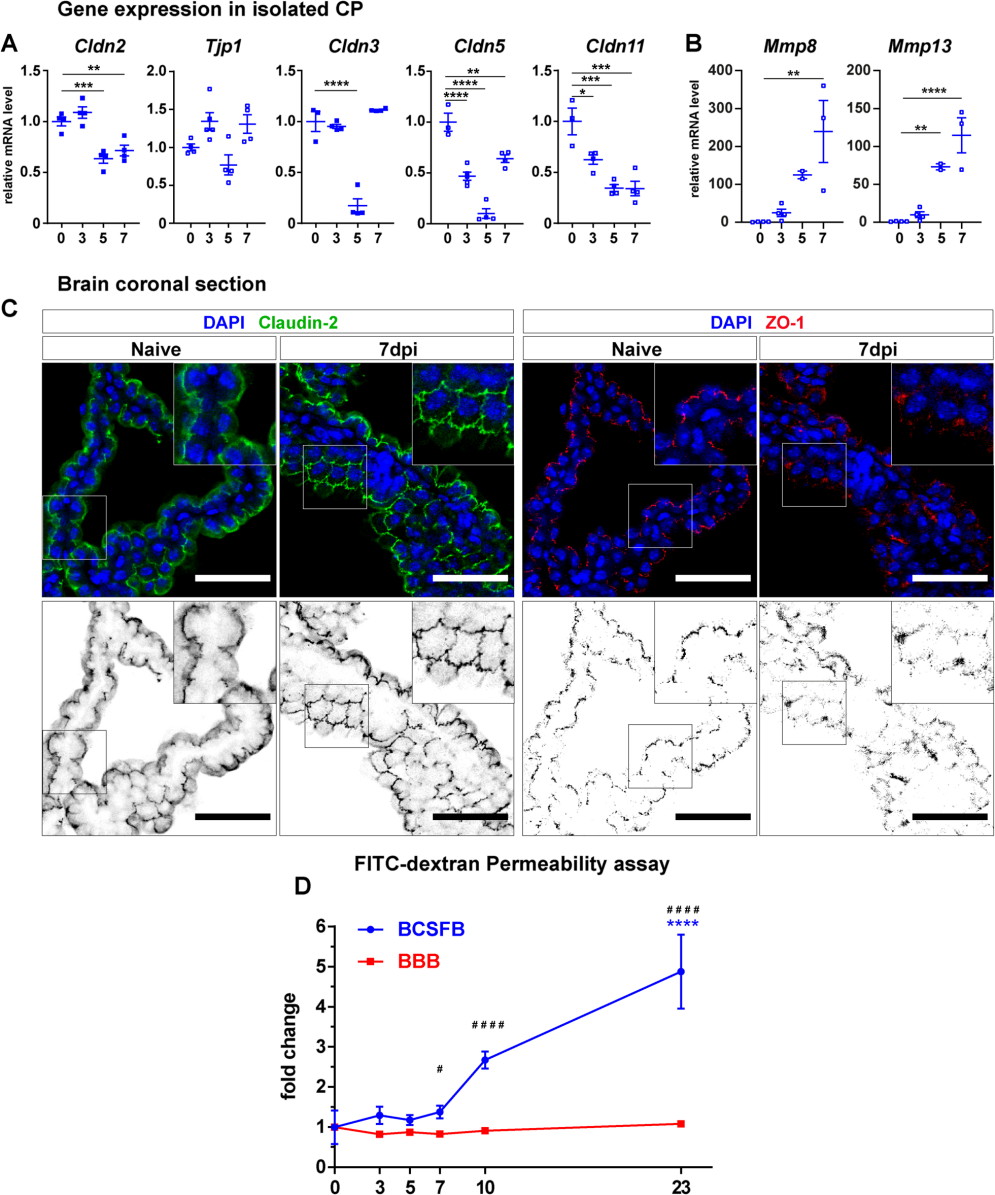
Dysregulated tight junctions in the CP affect BCSFB function during early infection. **A** Tight junctions and **B** MMP-8, and MMP-13 expression analysis (RT-PCR) of total RNA isolated from CP at 3, 5 and 7 dpi. Data show individual values and mean ± SEM, *n* = 2–5, *p < 0.05, **p < 0.01, ***p < 0.001, ****p < 0.0001 (one-way ANOVA, with Dunnett’s correction). **C** Immunofluorescence of coronal brain sections stained for identification of Claudin-2 (green) and ZO-1 (red) tight junctions from naïve and 7 dpi mice. White squares identify the regions of interest shown in higher magnification in the white upper squares. Lower panels indicate the respective inverted separated channels for each staining without DAPI to visualize the protein disturbances. Scale bars = 50 µm. **D** Functional FITC-dextran permeability assay comparing the leakage of the BCSFB (blue) versus BBB (red) throughout the course of infection. Data show mean ± SEM (*n* = 5) for each time-point analyzed. ****p < 0.0001, ^#^*p* < 0.05, ^####^*p* < 0.0001. # indicates significant difference between BCSFB and BBB from the same time-point (multiple *t*-test), and * indicates significant differences between day 0 and the time-point being analyzed from the same barrier type (one-way ANOVA, with Dunnett’s correction)

TJ molecules constitute the main components of the paracellular barrier established by CP epithelial cells, therefore they assume an essential role determining cellular and molecular compounds delivered from blood into CNS [25]. To investigate the outcome of *T. gondii* infection at the BCSFB, we analyzed the expression of TJs in isolated CP tissue over the course of infection. Claudins form the majority group of the integral membrane TJ proteins, which establish complex interactions with intracellular linker proteins like zonula occludens proteins (ZO, *Tjp*) [53]. Upon *T. gondii* infection at the CP, we detected lower expression levels of Claudin-2, -3, -5, and -11 compared to naïve group, suggesting a TJ alteration (Fig. 4A). Moreover, the breakdown of epithelial barriers has been associated with increased activity of matrix metalloproteinases (MMPs), which were indicated to be detrimental for BCSFB integrity during neuroinflammation [33]. Here, infected CP tissue displayed a robust upregulation of MMP-8 and MMP-13 (Fig. 4B), pointing towards a potential involvement of those endopeptidases in the epithelial barrier perturbation upon parasite invasion of the BCSFB. Taken together, the expression levels of claudins and MMPs in the infected CP suggested a barrier disruption. Indeed, immunostaining for Claudin-2 on brain sections showed ruffled-shape irregularities on the contour of CP epithelial cells in infected mice, instead of the smooth and continuous staining pattern depicted in naïve CP (Fig. 4C) [see Additional file 8]. In addition, ZO-1 staining indicates a grade of erasure in the infected CP, interestingly depicted in the same area of Claudin-2 morphological alteration. Finally, we determined whether altered TJ and MMP expression patterns affected CP barrier function. For this purpose, a FITC-dextran permeability assay was performed to assess barrier function in vivo. The extravasation of the fluorochrome-conjugated polysaccharide was quantified in the CSF for CP integrity, and in the remaining brain homogenate for BBB integrity (Fig. 4D). In contrast to the BBB, BCSFB permeability increased over time. A significant difference between both compartments was first visible at 7 dpi and further increased from 10 to 23 dpi. BBB vascular alterations were not depicted [see Additional file 9]. Thus, our results reveal a continuous increase in BCSFB permeability, suggesting long-standing detrimental effects of *T. gondii* infection on CP barrier integrity. Altogether, *T. gondii* infection results in TJs disturbances in the CP, with possible involvement of MMP-8 and MMP-13, culminating in functional loss and increased permeability of the BCSFB.

### Response of choroid plexus epithelial cells to *T. gondii* in vitro

**Fig. 5:**
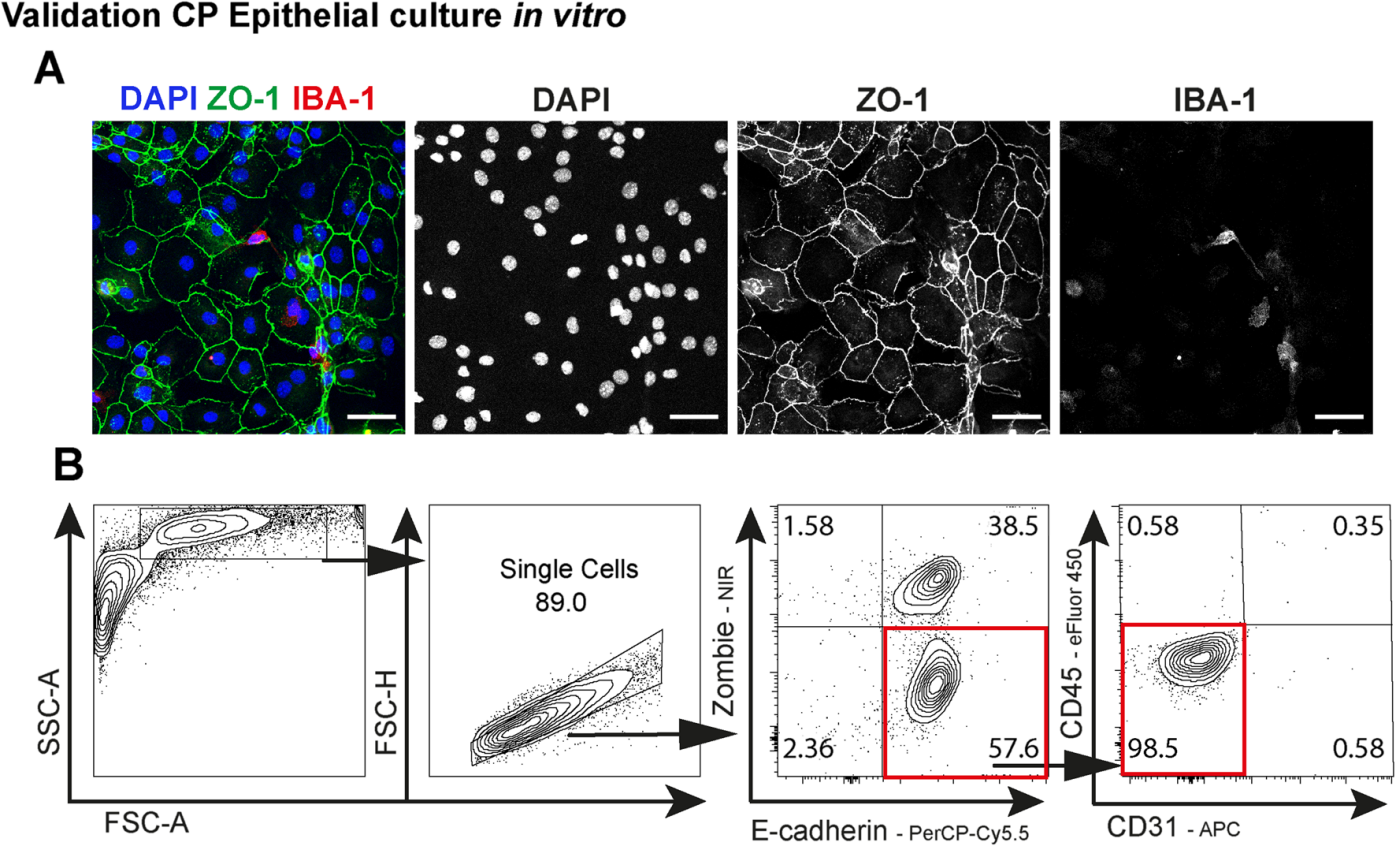
Validation of CP epithelial cell culture in vitro. **A** Representative image of cultured choroid plexus epithelial cells stained for the tight junction ZO-1. The myeloid cell marker IBA-1 was utilized as control for immune cell contamination. **B** Flow-cytometric validation of cultured epithelial cells, identified as E-cadherin^ +^ CD45^−^CD31 ^−^. Numbers represent the frequency from parent population

**Fig. 6:**
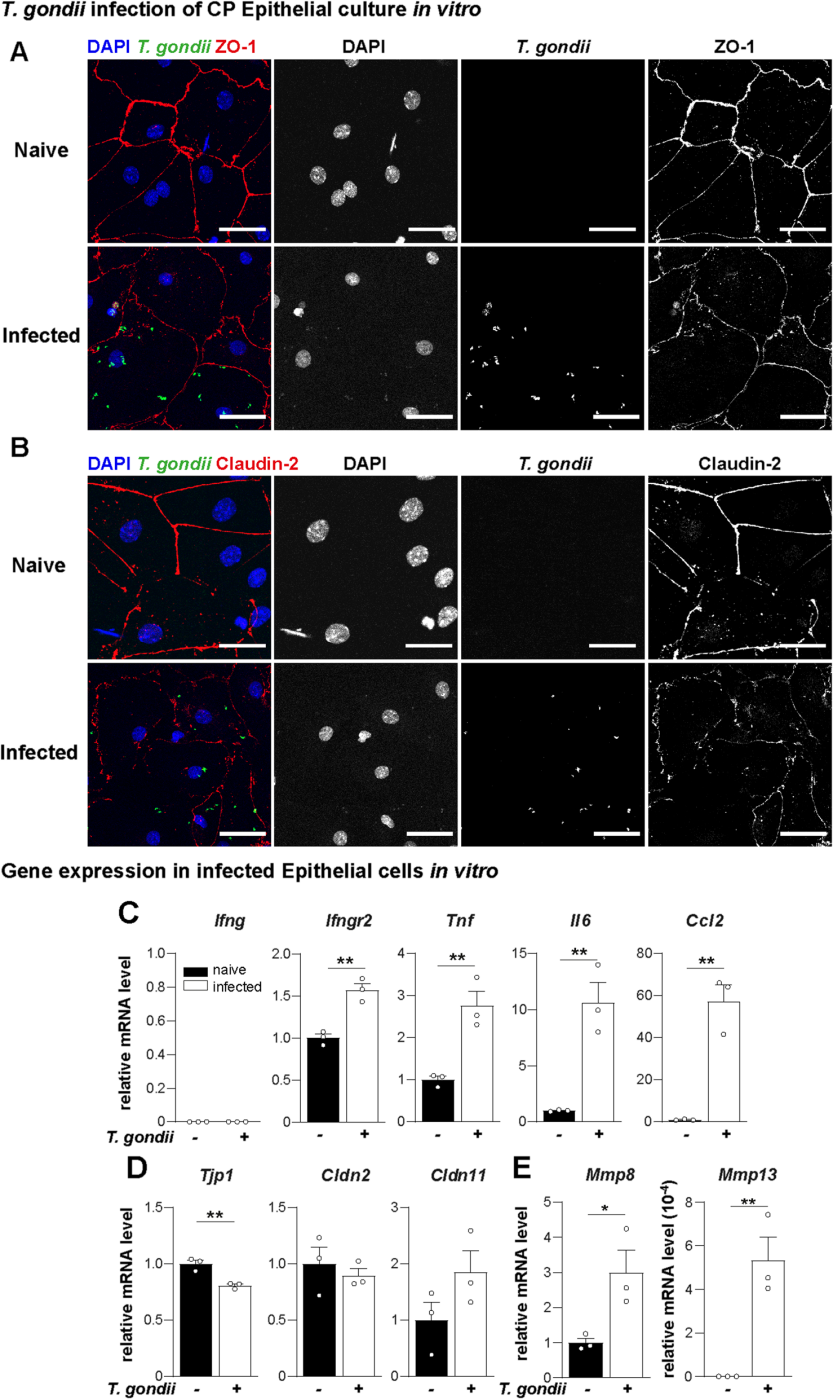
Epithelial CP cells response against *T. gondii* in vitro. **A**, **B** Primary cultures of CP epithelial cells were infected for 6 h with reporter *T. gondii* type II PRU-GFP tachyzoites at MOI = 5, and controls remained non-infected. Cultivated cells were immune-stained for detection of (**A**) ZO-1 (red) and (**B**) Claudin-2 (red). DAPI stained the nucleus (blue). GFP-reporter *T. gondii* (green). Scale bars = 50 µm. **C**–**E** Naïve and infected cultures were analyzed for gene expression of (**C**) cytokines and *Ifngr2*, (**D**) ZO-1 (*Tjp1*) and tight junctions, and (**E**) MMPs. Bar charts show individual mean values of triplicates from a representative experiment, and the mean + SEM (*n* = 3) for each time-point analyzed. Data were normalized by naïve means, besides *Mmp13* which was not detectable on naïve samples *p < 0.05, **p < 0.01(Student’s *t*-test)

For a more detailed characterization of *T. gondii* infection effects in the CP epithelium, we developed an in vitro culture model for murine primary CP epithelial cells. First, we confirmed the ability of the cultivated cells to establish adhesion junctions and express ZO-1 (honeycomb shape) (Fig. 5A). In addition, cell cultures were largely devoid of IBA-1 ^+^ myeloid cells and contained mainly CD45^−^ CD31^−^ E-cadherin^ +^ epithelial cells (Fig. 5B). Subsequently, CP epithelial cells were cultured in the presence or absence of GFP ^+^ *T. gondii* and the expression of the TJ proteins ZO-1 and Claudin-2 were quantified (Fig. 6). ZO-1 (Fig. 6A) and Claudin-2 (Fig. 6B) showed a poor and non-continuous distribution, characterized by strand breaks and puncta upon infection, compared to naïve controls. Additionally, the gene expression analysis of the cultivated epithelial cells showed no expression of IFN-γ, but increased expression of IFN-γ-receptor (*Ifngr2*) upon infection. The tissue damage-associated cytokines TNF and IL-6 were also upregulated, and the chemokine CCL2, responsible for myeloid cell recruitment was about 60-fold higher on infected cultures (Fig. 6C). We also detected a discrete reduction of ZO-1 (encoded by *Tjp1*) expression upon infection, without change and in the levels of Claudin-2 and Claudin-11 (Fig. 6D). In agreement with (Fig. 4B), MMP-8 and MMP-13 showed a robust upregulation upon infection, suggesting that CP epithelial cells are relevant source of MMPs (Fig. 6E). Overall, these results demonstrate that epithelial cells from CP directly respond to *T. gondii* infection, and contribute to the local inflammatory response and BCSFB damage.

## Discussion

The pivotal role of the CP in the orchestration of neuroinflammation became evident in the recent years [49, 54–56], and distinct pathogens have been described exploiting the CP as gateway into CNS [35]. These microbes cross CNS barriers via paracellular entry, transcellular penetration, or via infected immune cells (“Trojan horse”) [57]. The same mechanisms have been proposed for *T. gondii* invasion of the BBB [23], but only few studies have explored the CP as a gateway for *T. gondii* [36].

Here we demonstrate that *T. gondii* was found in the CP at the onset of infection. In a model of reactivated TE, there was no evidence for the involvement of the CP in *T. gondii* dissemination [37, 38]. Whether parasites are able to translocate from blood to the CSF, via CP, and further infect CSF-inundated areas (e.g., brain, spinal cord) is still not fully understood. In fact, we have previously described the extent of spinal cord pathology in a model of experimental chronic toxoplasmosis, suggesting that parasites translocate into CSF while the infection progresses [58]. Currently, the lack of tools to specifically block parasite entry into one or another barrier presents a major challenge to unveil their respective contributions to the parasite invasion. Here, we propose that CP infection is the initial step to the development of the neuroinflammation, resulting in the disruption of the BCSFB. Evidences show that the collapse of this barrier and its regulatory mechanisms allow immune cells to enter the CNS and initiate neuroinflammatory diseases [27]. As the disease evolves, the parasite spread increases, *e.g.*, through circulation of infected monocytes which simultaneously can cross the CNS via transposition of BBB and CP. It is likely that the higher parasite burden on BMV at 7 dpi indicate parasite shuttling to small CNS capillaries due to infected embolized monocytes, as suggested by previous studies [59].

Our findings suggest that the activation of the CP vasculature is involved in the orchestration of the anti-parasitic immune responses at early time points of infection. We observed that choroidal endothelial cells upregulated MHCII expression in response to *T. gondii* earlier than brain endothelial cells or microglia. Of note, brain endothelial cells have been described to serve as a replicative niche for *T. gondii* invasion of the CNS [7], but whether activated endothelial cells can control parasite replication in vivo is still unknown. Though, early MHCII presentation by CP endothelial cells suggests that they may play a role in antigen-presentation and interaction with T cells during *T. gondii* infection. They likely initiate a prompt recruitment of immune cells to the CP, orchestrating the onset of infection-induced neuroinflammation. In line with our findings, CP has been considered previously as a niche for T-cell proliferation and stimulation within the CNS [60]. Vascular and epithelial alterations at the CP, followed by other alterations like reduced choroidal synthesis, transport capacity, and CSF secretion were shown during aging, and were described to be intensified in Alzheimer’s disease [61]. Therefore, our data confirm the CP as an important interface to early pathogen interaction with endothelial cells, likely promoting damage recognition, antigen-presentation and immune cell recruitment.

Infections at the BCSFB has proven to promote CNS inflammation, inducing cytokines and chemokine secretion locally, and in the CSF, mediating immune cell activation and recruitment [62]. The CP senses CNS injury and rapidly responds to inflammation, upregulating the expression of adhesion molecules and chemokines receptors essential for leukocyte trafficking [50]. Upon the establishment of infection at the CP, we have found that *T. gondii* elicited early and local immune response at the BCSFB, with expression of pro-inflammatory cytokines, IFN-γ-inducible GTPases, and leukocyte trafficking molecules. In face of early detection of CD45^ +^ cells associated to *T. gondii* on the CP, we hypothesize that a transient burden of extracellular parasites in the blood likely reach the CP interface, infecting the choroidal endothelial cells and challenging the resident stromal macrophages and epithelial cells. Additionally, IFN-γ, TNF and IL-6 upregulation in the CP of infected mice might be due to the combined action of immune and epithelial cells, which showed elevated levels of gene expression after in vitro culture with *T. gondii*. This suggests a direct anti-parasitic, pro-inflammatory response of the CP epithelial cells. However, IFN-γ gene expression was only detectable in the CP in vivo, but not in CP epithelial cells co-cultured with *T. gondii*. This indicates that IFN-γ expression is restricted to immune cells. Whether they are CP-resident and/or recruited immune cells from the periphery remains to be clarified. IFN-γ-signaling through CP epithelium have been shown to tightly regulate cell recruitment into CNS [50], and probably will determine the immune cell dynamics through the CP upon *T. gondii* infection. Besides, we detected upregulation of IFN-γ-receptor (*Ifngr2*) on in vitro-infected CP epithelial cells, likely resulting in the increased expression of leukocyte trafficking genes CSF-1, CX3CL1, ICAM-1, CXCL9 and CXCL10, as previous described [50]. Moreover, we found CXCL9 more expressed in CP rather than in BMV through the first week of infection. Indeed, CXCL9 is known to induce T-cell activation and recruitment into the brain during cerebral toxoplasmosis [63]. Hereby, CXCL9 could be a differential trafficking molecule expressed by the CP to specific modulated the control of *T. gondii* infection.

Dysfunctional BCSFB is part of the pathophysiology leading to increased neuroinflammation [32]. At the BCSFB, the permeability is determined by TJ, specifically the Claudin-family proteins. Claudin-1, -2, -3 are highly expressed in the CP, and have been shown to be sensitive to inflammation [64–67]. In our analysis, Claudin-2 was downregulated and structurally disorganized within CP epithelial cells, displaying a ruffled shape upon infection. TJ ruffling frequently correlates with increased paracellular permeability caused by altered anchoring into actin filaments [68–70]. This anchoring is dependent on the scaffold protein ZO-1, which here displayed a grade of erasure in the infected CP. Accordingly, alterations of Claudins and ZO-1 in the CP have also been described in experimental autoimmune encephalomyelitis (EAE) and LPS-sepsis models [71, 72]. Of note, the infection of BCSFB by *Trypanosoma brucei* has indicated direct parasite interactions with Claudins of the CP, although the mechanisms of how they induce TJs opening is still not known [73, 74]. Still, the disruption of the TJ has been associated to the activity of MMPs enzymes implicated on BCSFB breakdown in neurodegenerative diseases and bacterial meningitis [29, 75]. We detected increased expression levels of the MMP-8 and MMP-13 on the infected CP, with both upregulated on in vitro CP epithelial cultures after *T. gondii* infection. Indeed, previous studies have described MMP-8 contribution to BCSFB leakage, and MMP-13 association with TJ dysregulation [31, 76].

Importantly, here we discovered that the CP damage extended to the chronic phase of experimental murine toxoplasmosis, presented by increased BCSFB permeability, which correlates to our previous findings on neuronal impairment during chronic toxoplasmosis [18, 20, 39, 77]. Therefore, we propose that the substantial change on the CP permeability has detrimental implications for CSF composition and impairment of the CNS drainage. Both aspects may contribute to the increased neuroinflammation and subsequent neuronal damage during toxoplasmosis.

## Conclusion

In summary, we detected that the CP is initially infected early after the onset of *T. gondii* infection, where next to CP endothelial cells also immune cells are targeted, determining a rapid inflammatory response and loss of BCSFB integrity and functionality. These processes are likely driven by TJ disturbance of Claudins within CP epithelial cells and by MMP activity. Thus, dysfunctional BCSFB may early enhance and further contribute to *T. gondii*-induced neuroinflammation, therefore emerging as a crucial target for new therapeutic approaches in cerebral toxoplasmosis.

## Supplementary Information

**Additional file 1: Table S1.** Oligonucleotide primers used for qPCR and RT-qPCR.

**Additional file 2. **Detection of *T. gondii* on BMV, brain with removed CP and Spinal cord. Animals were infected *i.p.* with 2 cysts of *T. gondii* type II ME49. CPs were removed and the remaining brain tissue was processed for isolation of BMVs. (A) BMVs from 7 dpi were stained to identify pericytes (PDGFRβ), *T. gondii* (SAG1), and the tight junction ZO-1. White square area is shown in higher magnification on the right image, and the yellow arrow points to the disseminated signal for *T. gondii*. (B) Parasite burden in spleens of infected mice at 3, 5 and 7 dpi. The analysis was performed based on the presence of B1 gene of *T. gondii* (TgB1) normalized to the murine gene *Asl*. Data were normalized to the mean values of 3 dpi, and bar charts show individual values of a representative experiment, and mean + SEM, *n* = 4.*p < 0.05 (multiple t-test, with Holm-Sidak correction). (C) Brains were isolated, CP removed, and remaining total brain homogenate was processed for parasite detection. (D) Spinal cords from infected mice were also used to quantify parasite burden. The analysis was performed based on expression of *Sag1* gene normalized to *Hprt* expression. Bar charts show individual values of a representative experiment, and mean + SEM, *n* = 4 (multiple t-test, with Holm-Sidak correction).

**Additional file 3. **Detection of *T. gondii* in the CP and brain. Animals were infected *i.p.* with 2 cysts of *T. gondii* type II ME49. The brains were isolated, and coronal sections or CP whole mount were immune-stained with anti-SAG1 (light blue), anti-CD31 (red), anti-Ecadherin (green) and DAPI (dark blue). (A) Confocal image of parasite detection on endothelial cells at 7 dpi. (B) Magnified region of interested previously identified by white square. (C) Parasites identified in the brain cortex, and white square region is magnified in (D). (E) Parasites on CP and adjacent brain ventricular areas at 7 dpi. (F) CP whole tissue mount from animals infected *i.p.* with 1 × 10^5^ *T. gondii* type II PRU-tdTomato tachyzoites, showing detection of parasites inside immune and endothelial cells at 3 dpi.

**Additional file 4. **IBA-1 staining and morphological identification of CP resident macrophages. Brain coronal sections of naïve animals were stained for identification of CP resident macrophages (elongated cells, red) indicated by yellow arrow. White squares indicate magnified region of interested. Scale bars = 50 µm.

**Additional file 5. **Alternative surface marker for identification of endothelial cells. Single cells suspensions obtained from CP (A-D), brain (E–H), liver (I-K) and lymph nodes (L-M) were digested and processed under the same conditions, and additional surface markers gp38 and VE-cadherin were used to verify identification of endothelial cells. Dot plots and numbers are from a representative sample. Bar charts represent the frequency in % of cells from parent population. Data represent individual values and mean ± SEM, *n* = 3.

**Additional file 6. **MHCII expression by microglia and CP epithelial cells. Single cells suspensions were discriminated based on FSC-SSC parameters, singlets, and viable cells (Zombie NIR negative). (A) Brain cells were gated and identified as microglia (CD11b^ +^ CD45^int^), CD45^ +^ immune cells, and double negative (DN) cells. (B) Choroid plexus cells were first divided in two main populations based on FSC-SSC. Bigger, viable cells were first defined as CD45^−^CD31^−^ then positive for the CP epithelial cell marker TTR (transthyretin). Smaller, viable cells were gated as CD45^ +^ CD31^−^ immune cells, and CD45^−^CD31^ +^ endothelial cells. (C) Representative contour plots showing microglia and (D) CP epithelial cells MHCII expression at 7 dpi. Bar charts represent the frequency in % of cells from parent population, and MFI values of MHCII expression levels. Data represent individual values and mean ± SEM, *n* = 5, **p < 0.01, ***p < 0.001, ****p < 0.0001 (one-way ANOVA, with Tukey’s correction).

**Additional file 7. **Complementary gene expression analysis of CP and BMVs. RT-PCR of total RNA from isolated tissue, for (A) MHCII expression on CP, (B, C) expression of interferon-beta-1 (*Ifnb1*) and macrophage colony-stimulating factor (*Csf1*), respectively, in isolated CP and BMV. (D, E) Expression of fractalkine (*Cx3cl1*) and intercellular-adhesion-molecule-1 (*Icam1*) on CP. Data show individual values and mean + SEM, *n* = 3–5, *p < 0.05, **p < 0.01, ***p < 0.001, ****p < 0.0001 (A, D, E, one-way ANOVA with Tukey’s correction; B, C, one-way ANOVA with Dunnett’s correction).

**Additional file 8. **Complementary staining of Claudin-2 in the CP. Brain coronal sections of naïve and 7 dpi animals were stained for identification of morphological alterations of Claudin-2 (green) in CP epithelium. Scale bars = 50 µm.

**Additional file 9. **Complementary BBB evaluation of VE-cadherin and ZO-1 upon infection. Animals were infected *i.p.* with 1 × 10^5^ *T. gondii* type II PTG-GFP tachyzoites (green). The brains were isolated at 7 dpi, and coronal sections were immune-stained for identification of VE-cadherin and ZO-1 tight junctions (red). Cortical and hippocampal areas were imaged. White squares identify the regions of interest shown in higher magnification.

## Data Availability

The datasets used and/or analyzed during the current study are available from the corresponding author on reasonable request.

## Abbreviations

AF488: Alexa Fluor 488
AF555: Alexa Fluor 555
AF594: Alexa Fluor 594
AF647: Alexa Fluor 647
ANOVA: Analysis of variance
BBB: Blood–brain barrier
BCSFB: Blood–cerebrospinal fluid barrier
BMV: Brain microvessels
CCL2: Chemokine (C–C motif) ligand 2
CD: Cluster of differentiation
CNS: Central nervous system
CP: Choroid plexus
CP-BAMS: Choroid plexus border-associated macrophages
CSF: Cerebrospinal fluid
CX3CL1: Chemokine (C-X3-C motif) ligand 1
CXCL10: Chemokine (C-X-C motif) ligand 10
CXCL9: Chemokine (C-X-C motif) ligand 9
DAAD: Deutscher Akademischer Austauschdienst
DAPI: 4′,6-Diamidino-2-phenylindole
DFG: Deutsche Forschungsgemeinschaft
DMEM: Dulbecco’s modified Eagle medium
DNA: Deoxyribonucleic acid
DNAse I: Deoxyribonuclease type I
dpi: Days post-infection
EDTA: Ethylenediamine tetraacetic acid
EGF: Epidermal growth factor
FACS: Fluorescence-activated cell sorting
FBS: Fetal bovine serum
FITC: Fluorescein isothiocyanate
Gbp2b: Guanylate binding protein 1
GFP: Green fluorescent protein
HBSS: Hank’s balanced salt solution
HEPES: 4-(2-Hydroxyethyl)-1-piperazine ethanesulfonic acid
HFF: Human foreskin fibroblasts
IBA-1: Ionized calcium binding adaptor molecule 1
ICAM-1: Intercellular adhesion molecule 1
Ifngr2: IFN-γ-receptor
IFN-β: Type I interferon-beta
IFN-γ: Interferon gamma
IgG: Immunoglobulin G
Igtp: Interferon gamma-induced GTPase
IL-6: Interleukin 6
Irgm1: Immunity-related GTPase family M protein 1
ITS: Insulin–transferrin–selenite
LPS: Lipopolysaccharides
M-CSF: Macrophage colony-stimulating factor
MHCII: Major histocompatibility complex type II
MMPs: Metalloproteinases
MOI: Multiplicity of infection
NEEA: Non-essential amino acids
NGS: Normal goat serum
OCT: Optimal cutting temperature
PBS: Phosphate-buffered saline
PCR: Polymerase chain reaction
PDGFRβ: Platelet derived growth factor receptor β
PFA: Paraformaldehyde
PLL: Poly-l-lysine
RNA: Ribonucleic acid
RT: Room temperature
RT-qPCR: Quantitative reverse transcription PCR
SAG1: Surface antigen 1
SEM: Standard error of the mean
*T. gondii*: *Toxoplasma gondii*
TE: Toxoplasma encephalitis
Tjp1: Tight junction protein-1
TJs: Tight junctions
TNF: Tumor necrosis factor
TTR: Transthyretin
ZO-1: Zona occludens-1
