# Supplementary material for: Immune response and pathogen invasion at the choroid plexus in the onset of cerebral toxoplasmosis
Source: J Neuroinflammation. 2022 Jan 13;19:17. doi: 10.1186/s12974-021-02370-1 (PMC8759173; doi:10.1186/s12974-021-02370-1)
Supplement: Supplementary file 1 — Additional file 1: Table S1. Oligonucleotide primers used for qPCR and RT-qPCR. [file 12974_2021_2370_MOESM1_ESM.docx]

**Supplementary table 1. Oligonucleotide primers used for *q*PCR and RT-*q*PCR**

| **SYBR Green** | | | | |
| --- | --- | --- | --- | --- |
|  | **GenBank Access Number** | | **Oligonucleotide Sequences** | |
|  |  |  | **Sense** | **Antisense** |
| **Target genes** | | |  |  |
| *Asl* | NM_133768 | | TCTTCGTTAGCTGGCAACTCACCT | ATGACCCAGCAGCTAAGCAGATCA |
| *Cldn11* | NM_008770.3 | | AAATGGACGAACTGGGCTCC | AGAACGGAGGCAGCAATCAT |
| *Cldn2* | NM_016675.4 | | GACGGCTCCGTTTTCTAGATGC | TCGTTTGGCTGCTGCTCTTG |
| *Cldn3* | NM_009902.4 | | GCAAGGACTACGTCTGAGGG | ACTGTGTGTCGTCTGTCACC |
| *Csf1* | NM_007778.4 | | CCACATGATTGGGAATGGAC | GTAGCAAACAGGATCATCCA |
| *Cx3cl1* | NM_009142.3 | | ATGTGCGACAAGATGACCTCACGA | TTTCTCCTTCGGGTCAGCACAGAA |
| *Cxcl10* | NM_021274.2 | | AACTGCATCCATATCGATGAC | GTGGCAATGATCTCAACAC |
| *Cxcl9* | NM_008599.4 | | GAGTTCGAGGAACCCTAGTG | AACTGTTTGAGGTCTTTGAGG |
| *H2-Aα* | NM_010378.3 | | ACCGTGACTATTCCTTCCA | CAGGTTCCCAGTGTTTCAG |
| *Hprt* | NM_013556 | | GCTATAAATTCTTTGCTGACCTGCTG | AATTACTTTTATGTCCCCTGTTGACTGG |
| *Icam1* | NM_010493.3 | | AGATCACATTCACGGTGCTGGCTA | AGCTTTGGGATGGTAGCTGGAAGA |
| *Ifngr2* | NM_008338.4 | | TCCCACACCCATTCACAG | AGGTCCAACAGTAACATTCTC |
| *Mmp13* | NM_008607.2 | | TTTATTGTTGCTGCCCATGA | GGTCCTTGGAGTGATCCAGA |
| *Mmp8* | NM_008611.4 | | ATTCCCAAGGAGTGTCCAAGC | TGATTGTCATATCTCCAGCACTGG |
| *Sag1 / SRS29B** | 44.m00009 | | ATCGCCTGAGAAGCATCACTG | CGAAAATGGAAACGTGACTGG |
| *T.gondii* B1 gene |  | | GCATTGCCCGTCCAAACT | AGACTGTACGGAATGGAGACGAA |
| ***TaqMan*®** | | | | |
| **Target genes** | | **Gene Expression Assays** | | |
| *Ccl2* | | Mm00441242_m1 | | |
| *Cldn5* | | Mm00727012_s1 | | |
| *Gbp2b* | | Mm00657086_m1 | | |
| *Hprt* | | Mm01545399_m1 | | |
| *Ifng* | | Mm00801778_m1 | | |
| *Igtp* | | Mm00497611_m1 | | |
| *Il6* | | Mm00446190_m1 | | |
| *Irgm1* | | Mm00492596_m1 | | |
| *Tjp1* | | Mm00493699_m1 | | |
| *Tnf* | | Mm00443258_m1 | | |
