# Supplementary figures and images for: Immune response and pathogen invasion at the choroid plexus in the onset of cerebral toxoplasmosis
Source: J Neuroinflammation. 2022 Jan 13;19:17. doi: 10.1186/s12974-021-02370-1 (PMC8759173; doi:10.1186/s12974-021-02370-1)

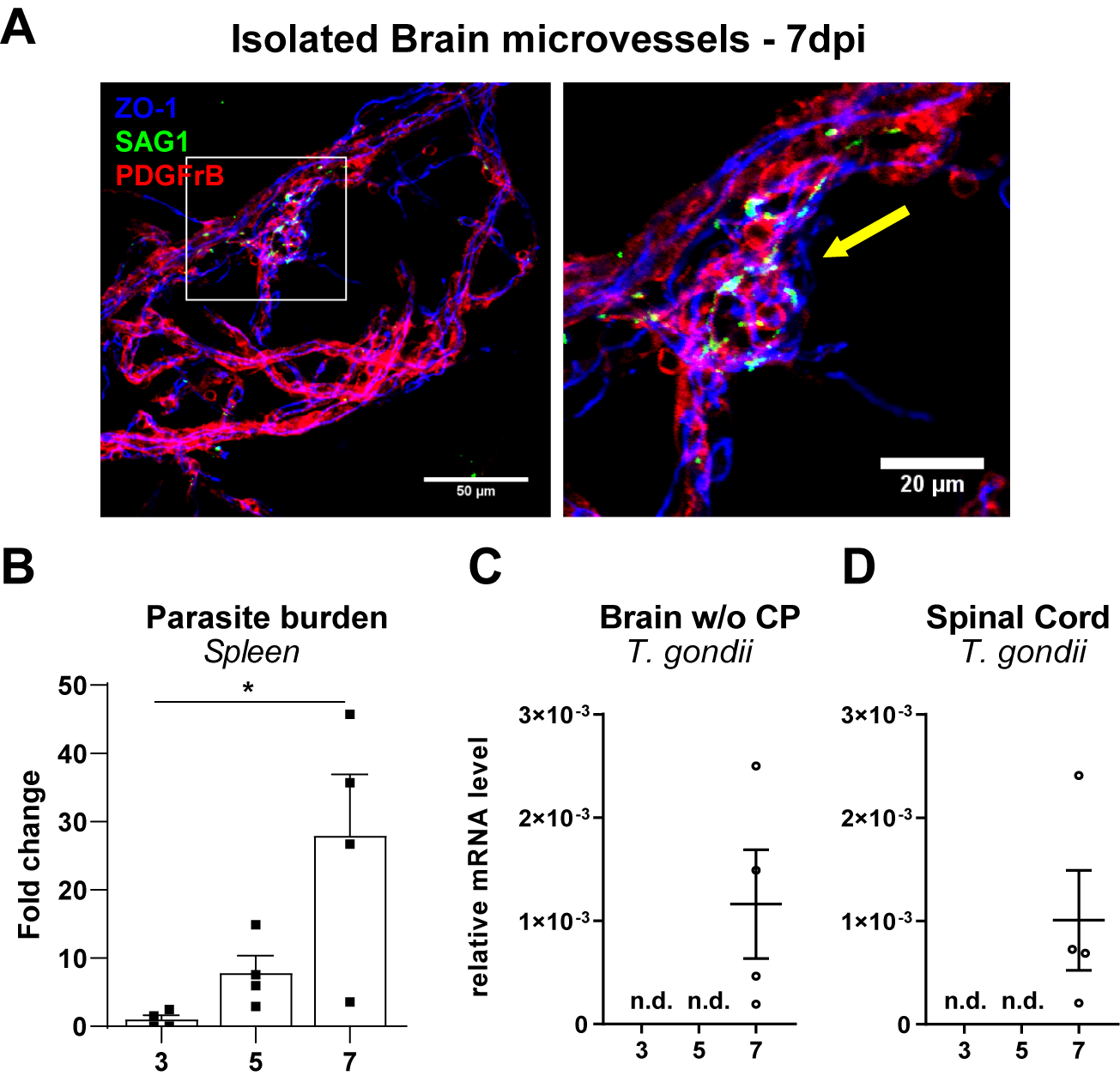

Supplement: Supplementary file 2 — Additional file 2. Detection of T. gondii on BMV, brain with removed CP and Spinal cord. Animals were infected i.p. with 2 cysts of T. gondii type II ME49. CPs were removed and the remaining brain tissue was processed for isolation of BMVs. (A) BMVs from 7 dpi were stained to identify pericytes (PDGFRβ), T. gondii (SAG1), and the tight junction ZO-1. White square area is shown in higher magnification on the right image, and the yellow arrow points to the disseminated signal for T. gondii. (B) Parasite burden in spleens of infected mice at 3, 5 and 7 dpi. The analysis was performed based on the presence of B1 gene of T. gondii (TgB1) normalized to the murine gene Asl. Data were normalized to the mean values of 3 dpi, and bar charts show individual values of a representative experiment, and mean + SEM, n = 4.*p < 0.05 (multiple t-test, with Holm-Sidak correction). (C) Brains were isolated, CP removed, and remaining total brain homogenate was processed for parasite detection. (D) Spinal cords from infected mice were also used to quantify parasite burden. The analysis was performed based on expression of Sag1 gene normalized to Hprt expression. Bar charts show individual values of a representative experiment, and mean + SEM, n = 4 (multiple t-test, with Holm-Sidak correction). [file 12974_2021_2370_MOESM2_ESM.tif]

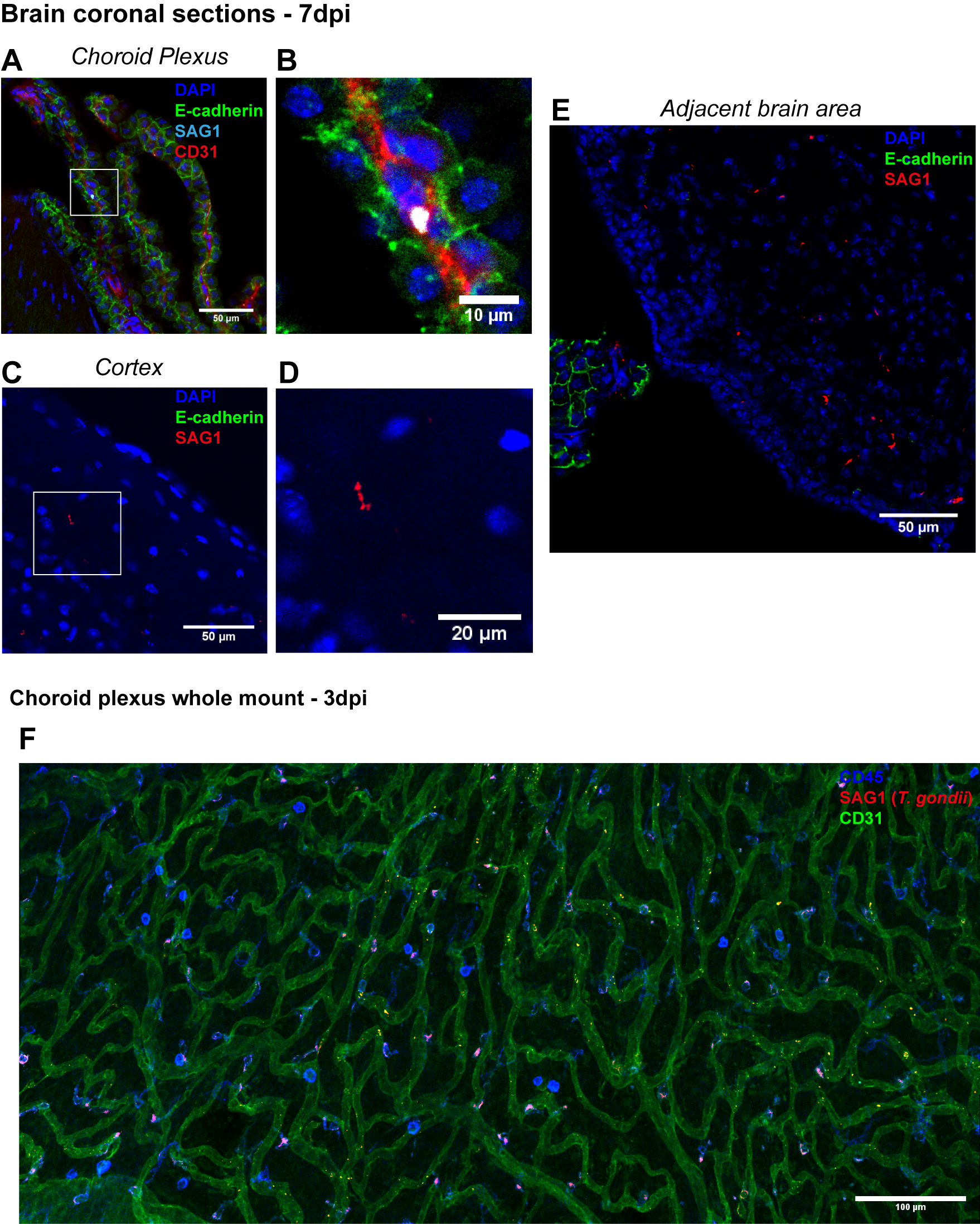

Supplement: Supplementary file 3 — Additional file 3. Detection of T. gondii in the CP and brain. Animals were infected i.p. with 2 cysts of T. gondii type II ME49. The brains were isolated, and coronal sections or CP whole mount were immune-stained with anti-SAG1 (light blue), anti-CD31 (red), anti-Ecadherin (green) and DAPI (dark blue). (A) Confocal image of parasite detection on endothelial cells at 7 dpi. (B) Magnified region of interested previously identified by white square. (C) Parasites identified in the brain cortex, and white square region is magnified in (D). (E) Parasites on CP and adjacent brain ventricular areas at 7 dpi. (F) CP whole tissue mount from animals infected i.p. with 1 × 10^5^ T. gondii type II PRU-tdTomato tachyzoites, showing detection of parasites inside immune and endothelial cells at 3 dpi. [file 12974_2021_2370_MOESM3_ESM.tif]

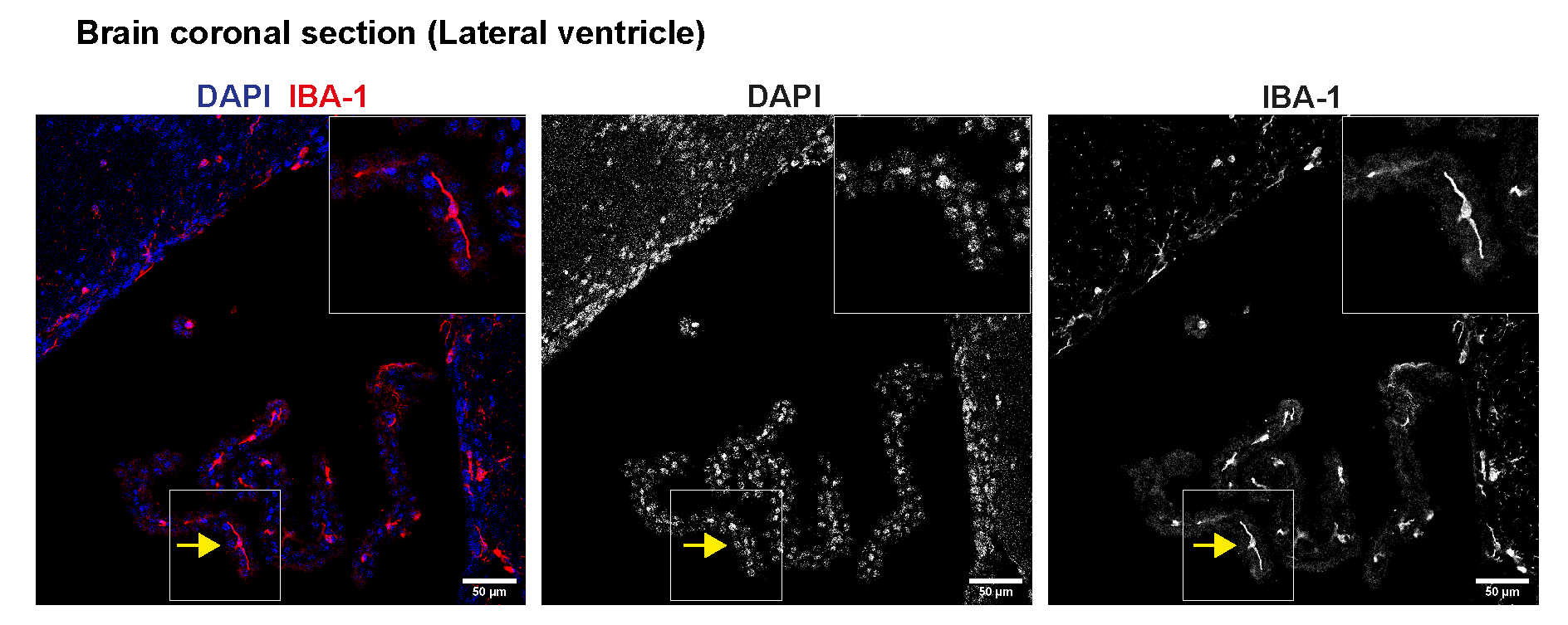

Supplement: Supplementary file 4 — Additional file 4. IBA-1 staining and morphological identification of CP resident macrophages. Brain coronal sections of naïve animals were stained for identification of CP resident macrophages (elongated cells, red) indicated by yellow arrow. White squares indicate magnified region of interested. Scale bars = 50 µm. [file 12974_2021_2370_MOESM4_ESM.tif]

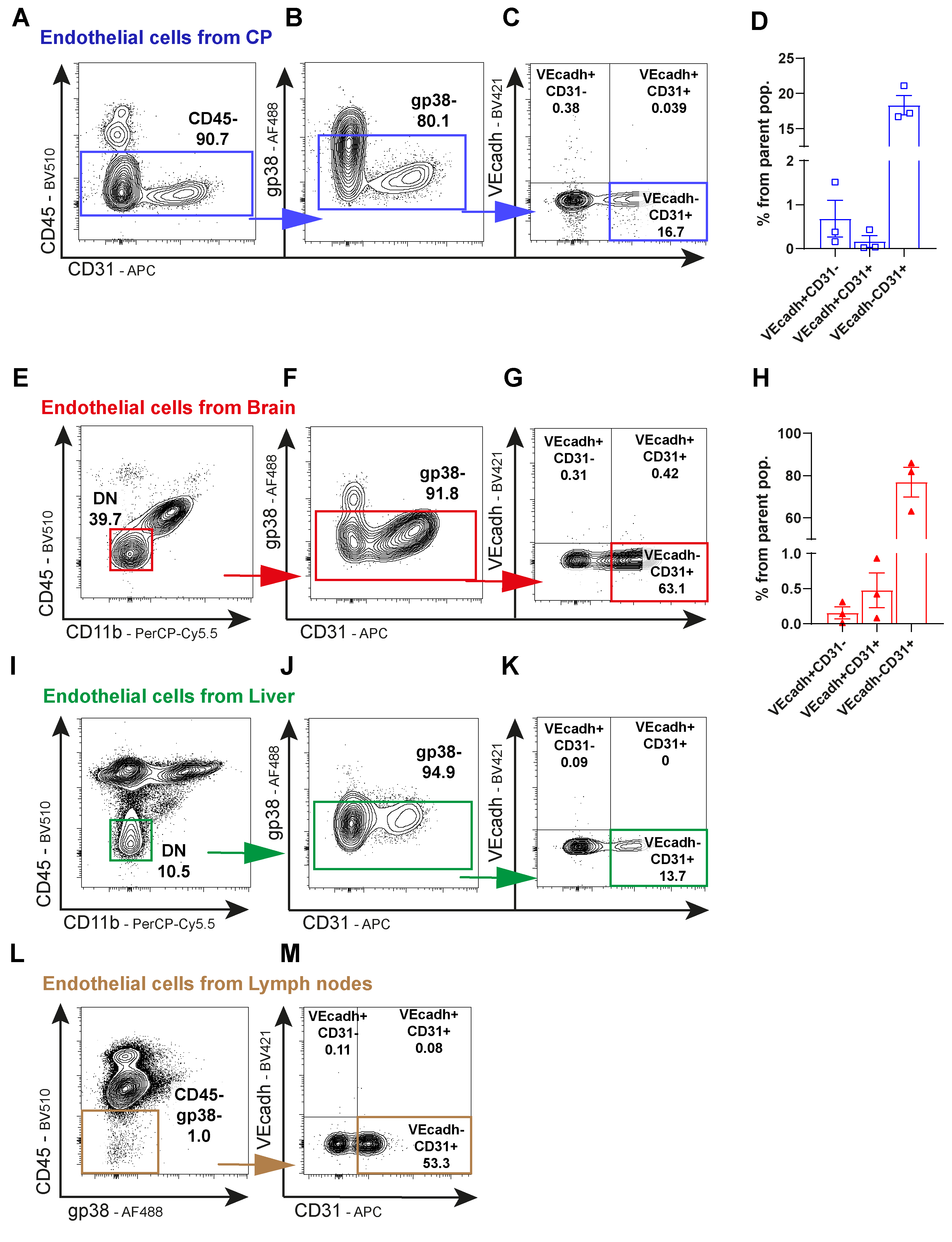

Supplement: Supplementary file 5 — Additional file 5. Alternative surface marker for identification of endothelial cells. Single cells suspensions obtained from CP (A-D), brain (E–H), liver (I-K) and lymph nodes (L-M) were digested and processed under the same conditions, and additional surface markers gp38 and VE-cadherin were used to verify identification of endothelial cells. Dot plots and numbers are from a representative sample. Bar charts represent the frequency in % of cells from parent population. Data represent individual values and mean ± SEM, n = 3. [file 12974_2021_2370_MOESM5_ESM.tif]

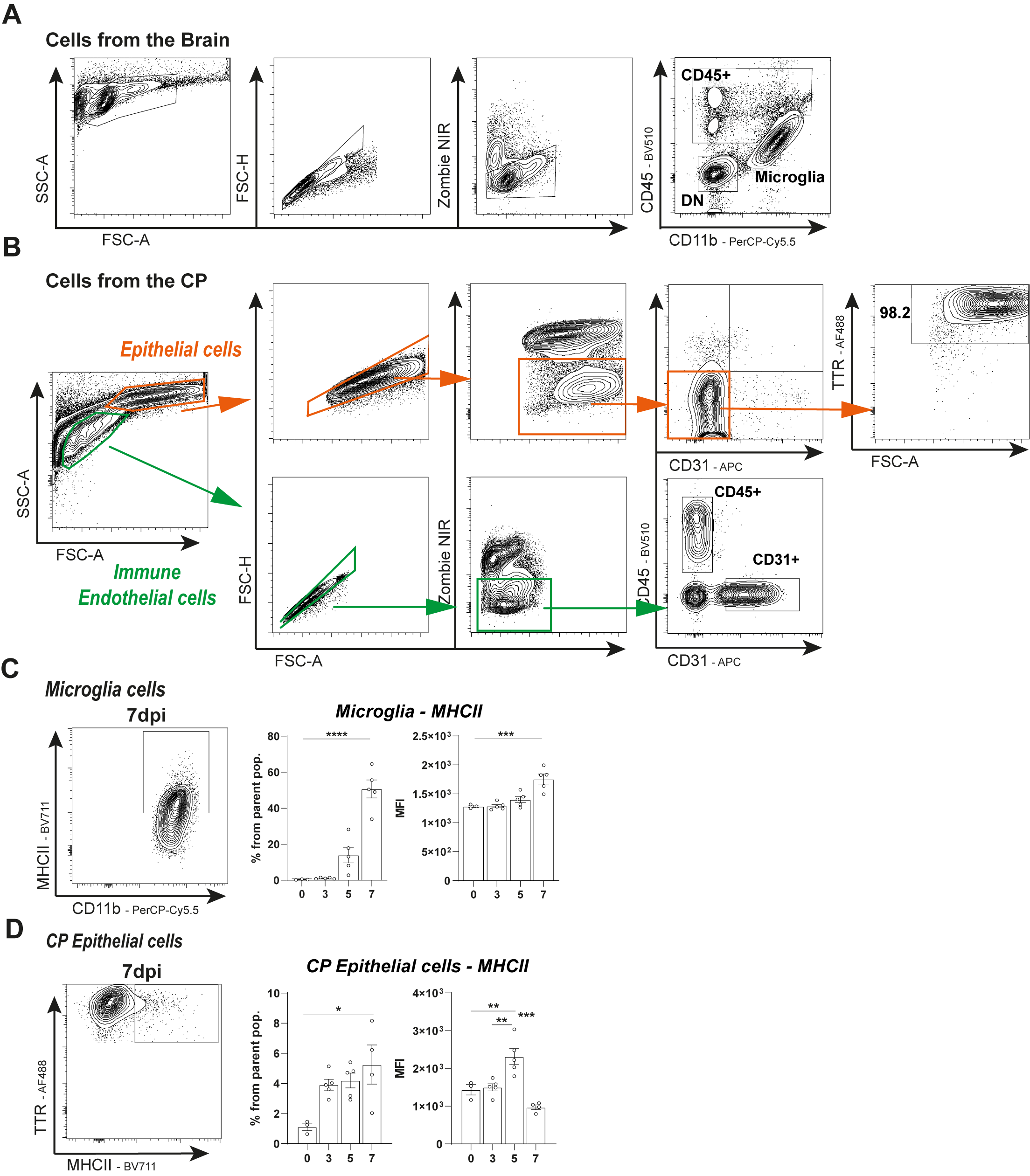

Supplement: Supplementary file 6 — Additional file 6. MHCII expression by microglia and CP epithelial cells. Single cells suspensions were discriminated based on FSC-SSC parameters, singlets, and viable cells (Zombie NIR negative). (A) Brain cells were gated and identified as microglia (CD11b + CD45int), CD45 + immune cells, and double negative (DN) cells. (B) Choroid plexus cells were first divided in two main populations based on FSC-SSC. Bigger, viable cells were first defined as CD45−CD31− then positive for the CP epithelial cell marker TTR (transthyretin). Smaller, viable cells were gated as CD45 + CD31− immune cells, and CD45−CD31 + endothelial cells. (C) Representative contour plots showing microglia and (D) CP epithelial cells MHCII expression at 7 dpi. Bar charts represent the frequency in % of cells from parent population, and MFI values of MHCII expression levels. Data represent individual values and mean ± SEM, n = 5, **p < 0.01, ***p < 0.001, ****p < 0.0001 (one-way ANOVA, with Tukey’s correction). [file 12974_2021_2370_MOESM6_ESM.tif]

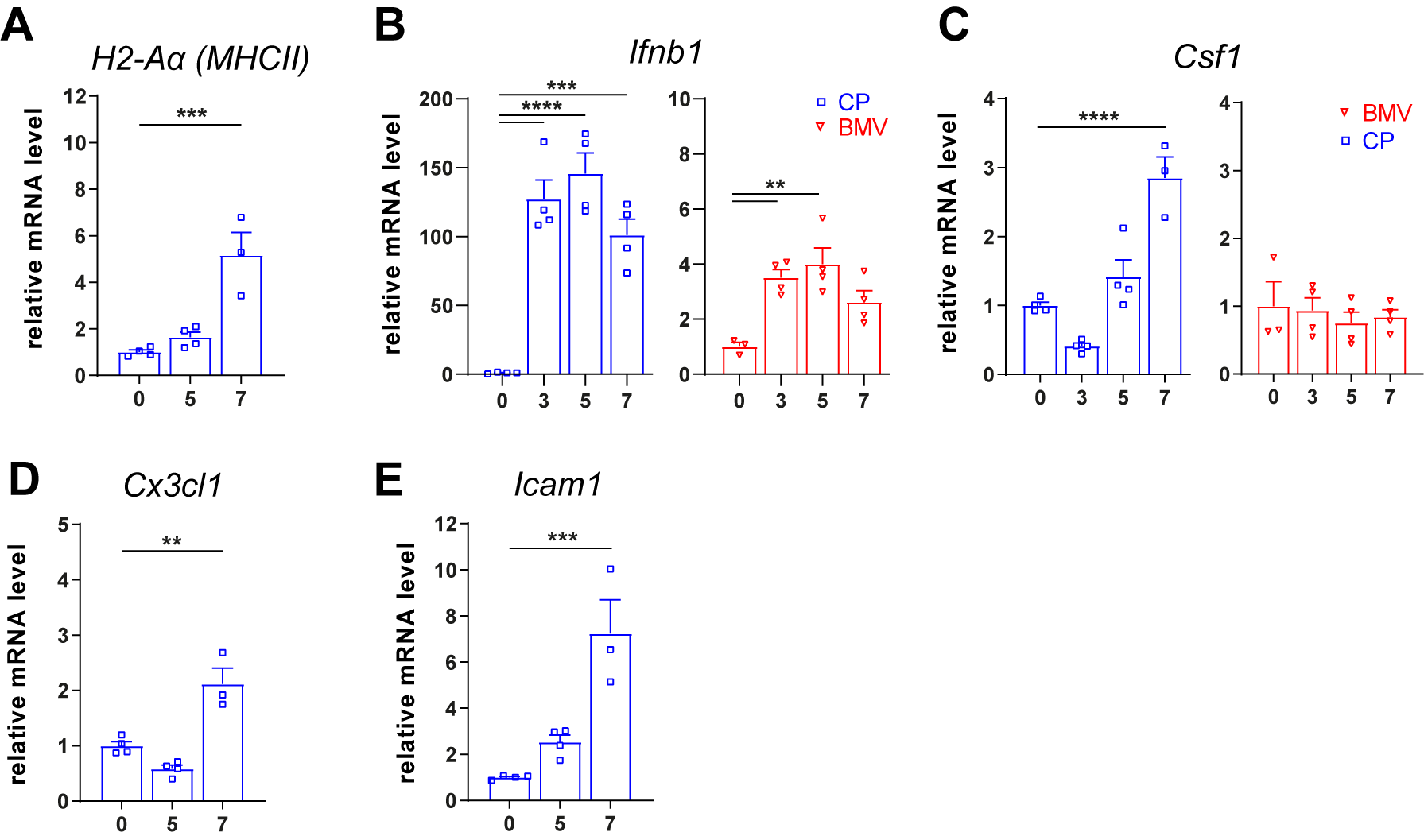

Supplement: Supplementary file 7 — Additional file 7. Complementary gene expression analysis of CP and BMVs. RT-PCR of total RNA from isolated tissue, for (A) MHCII expression on CP, (B, C) expression of interferon-beta-1 (Ifnb1) and macrophage colony-stimulating factor (Csf1), respectively, in isolated CP and BMV. (D, E) Expression of fractalkine (Cx3cl1) and intercellular-adhesion-molecule-1 (Icam1) on CP. Data show individual values and mean + SEM, n = 3–5, *p < 0.05, **p < 0.01, ***p < 0.001, ****p < 0.0001 (A, D, E, one-way ANOVA with Tukey’s correction; B, C, one-way ANOVA with Dunnett’s correction). [file 12974_2021_2370_MOESM7_ESM.tif]

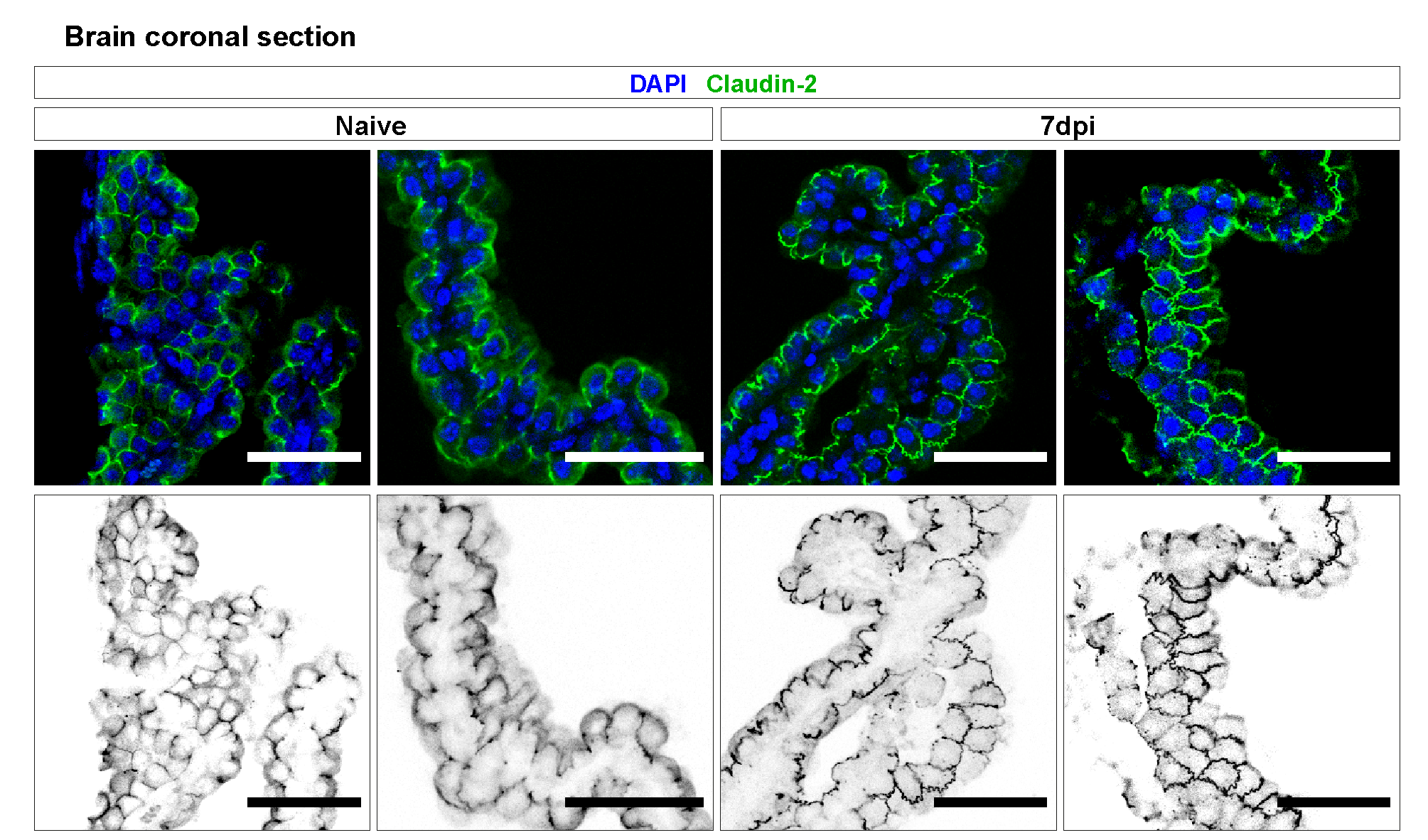

Supplement: Supplementary file 8 — Additional file 8. Complementary staining of Claudin-2 in the CP. Brain coronal sections of naïve and 7 dpi animals were stained for identification of morphological alterations of Claudin-2 (green) in CP epithelium. Scale bars = 50 µm. [file 12974_2021_2370_MOESM8_ESM.tif]

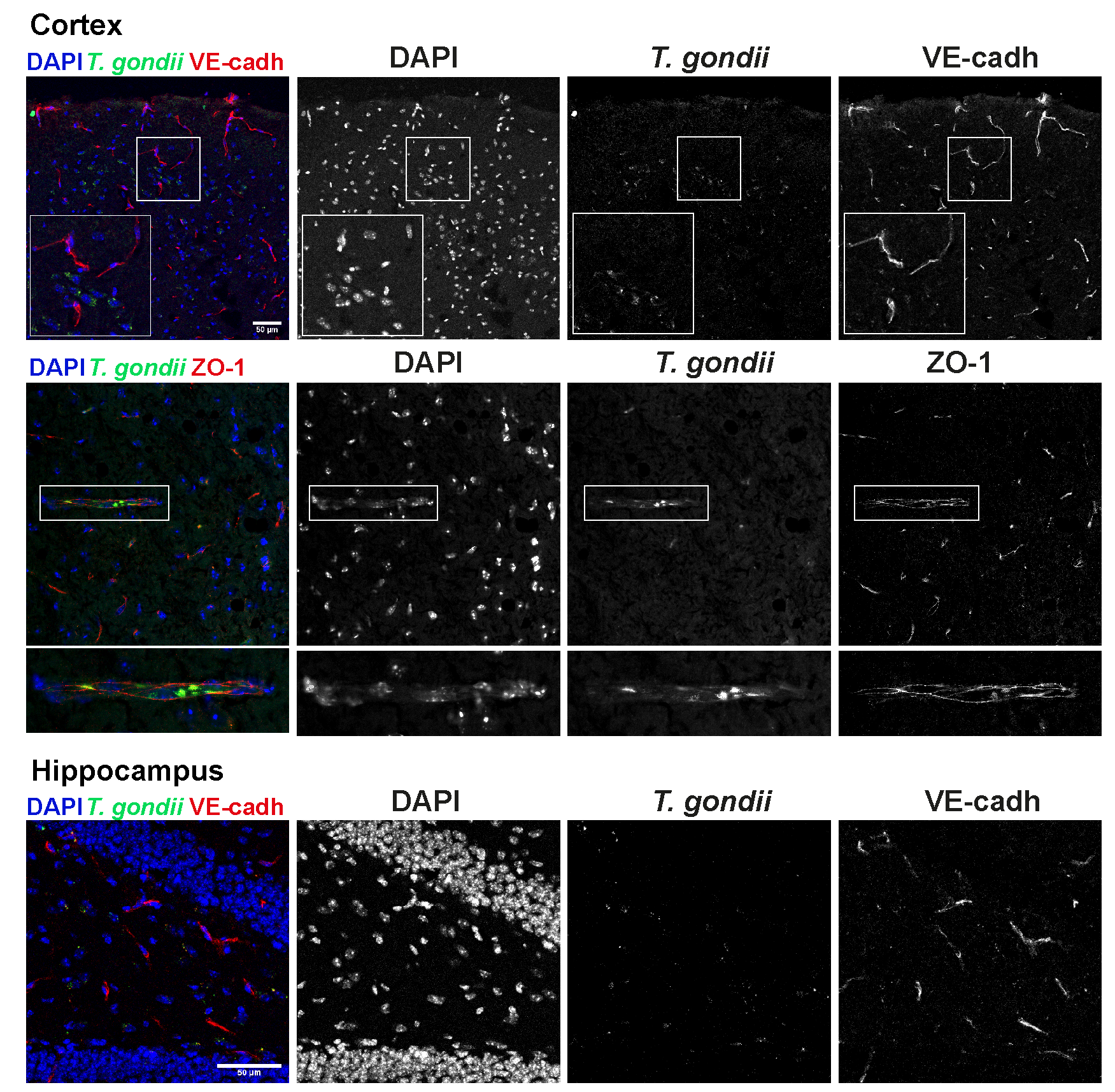

Supplement: Supplementary file 9 — Additional file 9. Complementary BBB evaluation of VE-cadherin and ZO-1 upon infection. Animals were infected i.p. with 1 × 10^5^ T. gondii type II PTG-GFP tachyzoites (green). The brains were isolated at 7 dpi, and coronal sections were immune-stained for identification of VE-cadherin and ZO-1 tight junctions (red). Cortical and hippocampal areas were imaged. White squares identify the regions of interest shown in higher magnification. [file 12974_2021_2370_MOESM9_ESM.tif]
